# Reconstructing the Phylogeny of *Corynebacteriales* while Accounting for Horizontal Gene Transfer

**DOI:** 10.1093/gbe/evaa058

**Published:** 2020-04-02

**Authors:** Nilson Da Rocha Coimbra, Aristoteles Goes-Neto, Vasco Azevedo, Aïda Ouangraoua

**Affiliations:** e1 Department of Computer Science, University of Sherbrooke, Quebec, Canada; e2 Programa Interunidades de Pós-graduação em Bioinformática, Universidade Federal de Minas Gerais, Belo Horizonte, Minas Gerais, Brazil

**Keywords:** phylogeny estimation, bacteria, horizontal gene transfer, *Corynebacterium*, *Mycobacterium*

## Abstract

Horizontal gene transfer is a common mechanism in Bacteria that has contributed to the genomic content of existing organisms. Traditional methods for estimating bacterial phylogeny, however, assume only vertical inheritance in the evolution of homologous genes, which may result in errors in the estimated phylogenies. We present a new method for estimating bacterial phylogeny that accounts for the presence of genes acquired by horizontal gene transfer between genomes. The method identifies and corrects putative transferred genes in gene families, before applying a gene tree-based summary method to estimate bacterial species trees. The method was applied to estimate the phylogeny of the order *Corynebacteriales*, which is the largest clade in the phylum *Actinobacteria*. We report a collection of 14 phylogenetic trees on 360 *Corynebacteriales* genomes. All estimated trees display each genus as a monophyletic clade. The trees also display several relationships proposed by past studies, as well as new relevant relationships between and within the main genera of *Corynebacteriales*: *Corynebacterium*, *Mycobacterium*, *Nocardia*, *Rhodococcus*, and *Gordonia*. An implementation of the method in Python is available on GitHub at https://github.com/UdeS-CoBIUS/EXECT (last accessed April 2, 2020).

## Introduction

One of the major discoveries in the 20th century is the bacterial production of antibiotics, which are useful in treating bacterial infections (Bister et al. 2004; Fair and Tor 2014). The ongoing evolution of bacteria, however, contributes to the appearance of new bacterial species, including new antibiotic-resistant pathogenic species (Fischbach and Walsh 2009). In terms of genome structure, bacterial species differ from each other in the content and arrangement of genes in their genomes, which results from genome rearrangement, gene duplication, gene loss, and horizontal gene transfer (HGT) events (Gogarten et al. 2002). In particular, HGT has been shown to be a primary force underlying antibiotic-resistance and virulent genes spreading in Bacteria (Ruiz et al. 2011; Zhi et al. 2017).

HGT is the transfer of genetic material through a process different from vertical inheritance (Soucy et al. 2015). The modules of genetic transfer are usually genes, but it was also shown that HGT can occur at the level of protein domains (Chan et al. 2009). The prevalence of HGT events in bacterial evolution limits the use of phylogenetic methods that assume only vertical inheritance evolutionary events. Traditionally, alignments of sequences of 16S rRNA genes have been used to estimate bacterial phylogenies and study bacterial diversity. This approach relies on the assumption that 16S rRNA genes constitute essential genes that are only vertically inherited. Several studies have, however, reported evidence for HGT of 16S rRNA genes (Yap et al. 1999; Schouls et al. 2003; Kitahara and Miyazaki 2013; Miyazaki et al. 2017). Moreover, the identification and classification bacterial species based solely on 16S rRNA genes often lead to errors in phylogenetic estimations (Rajendhran and Gunasekaran 2011). The reason could be the intragenomic heterogeneity in bacterial rRNA as well as the presence of mosaicism and multiple copies of 16S rRNA genes in genomes, which may result from HGT events (Klappenbach et al. 2000; Schouls et al. 2003). In this context, the main contribution of this work is a method for estimating bacterial phylogenies with sets of gene families but without assuming only vertical inheritance in the evolution of gene families.

Phylogenetic reconstruction usually relies on two steps: first, the identification of groups of orthologous sequences in genomes, and second, the construction of a tree explaining the evolution within orthology groups by vertical inheritance (Felsenstein 1985). Therefore, computing accurate orthology groups in the first step is a prerequisite for reconstructing accurate phylogenies in the second step. For the second step, phylogeny methods can be classified into three main approaches: alignment-based, gene order-based, and gene tree-based methods (Wolf et al. 2002).

Alignment-based methods infer the species tree based on a concatenation of multiple sequence alignments on the orthology groups. This approach has been widely used because it scales well to a large number of orthology groups and species (Rokas et al. 2003; Ciccarelli et al. 2006). Nonetheless, alignment-based methods do not allow for accounting for the impact of genome content and structure evolution in estimating species diversity (Saitou and Nei 1987). Another class of alignment-based methods are whole genome single-nucleotide polymorphism-based methods that start by removing signals from recombination and then build a species tree using whole genome alignments, single-nucleotide polymorphisms, and maximum-likelihood (ML) approaches (Castillo-Ramírez et al. 2012; Comas et al. 2013).

Gene order-based methods infer a species tree based on the difference between genomes in terms of gene content and arrangement (Sankoff and Blanchette 1998; Bourque et al. 2004; Belda et al. 2005). They allow for accounting for the evolution of gene content and arrangement as well as gene conservation or splitting. Gene order-based methods are suitable for reconstructing phylogenies of closely related species (Moret et al. 2013). Nevertheless, this approach is limited by the complexity in scaling up to large data sets and the lack of a well-defined model of gene order evolution (Moret et al. 2001).

Gene tree-based methods consist in using a set of gene trees—one for each orthology group—in order to estimate a species tree that could explain the evolution of gene families within the species tree (Suyama and Bork 2001). Such methods are currently in limited use in estimating bacterial phylogeny because they are very sensitive to the presence of erroneous genes in orthology groups caused by HGT events, leading to a disruption in the phylogenetic signals (Ravenhall et al. 2015). Thus, the detection and discarding of transferred genes from orthology groups is a prerequisite for using gene tree-based methods for estimating bacterial phylogeny.

Computational methods for HGT detection can be classified into two main approaches: parametric methods and comparative methods. Parametric methods are intragenomic, and exploit sequence composition changes along a genome sequence to infer putative HGT regions. Comparative methods are intergenomic and include alignment-based and phylogeny-based methods. Alignment-based methods—such as MobilomeFINDER (Ou et al. 2007)—make use of the alignment between closely related genomes to infer HGT. Phylogeny-based methods—such as NOTUNG (Chen et al. 2000)—exploit the inconsistencies between gene trees and species trees to infer HGT (Lerat et al. 2005; Ravenhall et al. 2015; Jeong et al. 2019). On the one hand, parametric methods—such as IslandPath-DIMOB (Bertelli and Brinkman 2018)—take advantage of relying solely on genome sequences by an intrinsic analysis. They achieve average recall rates with high precision rates. On the other hand, comparative methods are limited by their requirement of an accurate species tree, which, in turn, is challenging to build in the presence of HGT (Lasek-Nesselquist et al. 2012). Nevertheless, when a preliminary, partially resolved species tree is available, the congruence of a gene tree with this species tree can be used to correct the misclassification of some genes as transferred genes inferred by parametric methods.

**Fig. 1.**
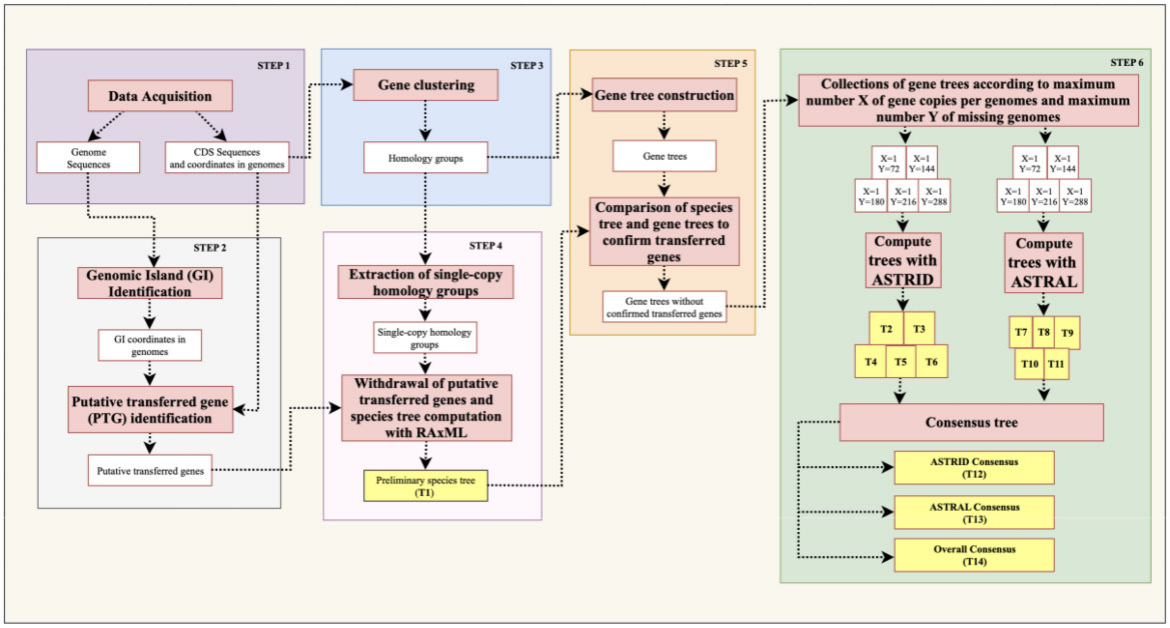
—Overview of the method, which consists of six steps.

This article presents a gene tree-based method accounting for HGT events in estimating bacterial phylogenies (fig. 1). After collecting the input data set, which consists of genome sequences with the locations of their coding DNA sequences (CDSs) representing genes (Step 1), the method starts by detecting putative transferred genes (PTGs) with a parametric method (Step 2). PTGs are identified using an intragenomic genomic island (GI) detection method in order to avoid the circular argument of detecting HGT using a species tree, and then removing HGT form gene trees to compute a species tree. In parallel, genes are clustered into homology groups based on their CDS similarities (Step 3). Subsequently, putative orthology groups, containing a single gene per genome, are used to build a preliminary, partially resolved species tree with an alignment-based phylogenetic method (Step 4). The preliminary species tree is used to correct misclassified PTGs in homology groups with a phylogenetic approach. The latter consists in comparing the gene tree of each homology group with the species tree in order to compare the phylogenetic position of PTG in the two trees. A PTG whose location induces no HGT in the reconciliation between the gene tree and the species tree is reclassified as a vertically inherited gene (Step 5). Lastly, the remaining transferred genes are removed from homology groups. The latter are used to build gene trees and the final species trees using phylogenetic gene tree-based methods (Step 6).

The pipeline was applied to estimate the phylogeny of *Corynebacteriales*, the largest clade of the phylum *Actinobacteria* in terms of number of available genomes. The input data set comprised 360 complete genome sequences obtained in Step 1, and the output consisted of 10 distinct phylogenetic trees on the 360 genomes that were estimated with 2 gene tree-based phylogenetic methods, ASTRID (Vachaspati and Warnow 2015) and ASTRAL II (Mirarab and Warnow 2015) in Step 6. The similarity of the estimated phylogenies was compared by computing the percentage of conserved clades between each pair of trees. The final phylogeny of *Corynebacteriales* was obtained by computing consensus trees using the majority-rule consensus (Retief 2000) in two phases. First, the collections of trees obtained with ASTRAL and ASTRID were reduced to two trees. Then, those two trees and the preliminary species tree from Step 4 were reduced to a single tree.

## Materials and Methods 

Figure 1 depicts the entire method used to estimate the phylogeny of *Corynebacteriales*. The details and rationale underlying each step in the method are described below.

### Step 1: Data Acquisition

Step 1 consists in acquiring the *Corynebacteriales* genome and gene data. All complete *Corynebacteriales* genome sequences were retrieved from the REFSEQ NCBI database, release 81 (Maglott et al. 2005). The CDSs and gene coordinates were subsequently extracted using the genome annotations.

### Step 2: Detecting GIs and Identifying PTGs

Identifying horizontally transferred genes in bacterial genomes is a prerequisite to computing a bacterial phylogenetic tree using gene tree-based phylogeny methods. Horizontally transferred genes can be located in GIs, which are large segments of DNA (10–200 kb) acquired by horizontal transfer (Langille et al. 2010). There are several approaches for detecting the GI regions in genomes. Some methods—such as MobilomeFINDER (Ou et al. 2007)—make use of a comparative genomics approach and identify GIs as deleted or inserted regions inferred by aligning closely related genomes. Other comparative genomics methods—such as NOTUNG (Chen et al. 2000)—identify HGT by detecting discordance between a gene tree and a species tree. Other methods, referred to as parametric, make use of a sequence composition approach that defines GIs as regions with dinucleotide (G + C) bias or codon usage bias containing associated mobility genes. Compared with parametric methods, comparative approaches have the advantage of being able to detect old HGT events despite the process of sequence homogenization undergone by old GI regions. They however require the availability of closely related genomes or a reliable species tree for the input genomes. Because neither closely related genomes for all genomes of the *Corynebacteriales* data set nor any reliable input species tree was available, GIs were detected with the parametric method IslandPath-DIMOB v1.0.0, which is currently the most accurate stand-alone method for GI prediction (Bertelli and Brinkman 2018) (recall rate of 46.9% and high precision rate of 87.4%). The default parameters of IslandPath-DIMOB were used. The genes contained in the detected GI regions were classified as PTGs. Note that, because of the recall rate of the method, there may be horizontally transferred genes located in GIs that were not detected by the method. They may also be transferred genes not detected by the method because they are not located in GIs. These undetected horizontally transferred genes are considered in Step 6 during the species trees construction.

### Step 3: Clustering of Genes into Homology Groups

The CDSs extracted in Step 1 were translated into protein sequences and clustered using Orthofinder1 (Emms and Kelly 2015). This protein clustering tool was chosen because of its high accuracy compared with other currently available gene clustering methods (Emms and Kelly 2015). Orthofinder solves gene length bias before constructing gene groups. An all-against-all BlastP with a stringent cutoff *e*-value of 10^−4^ was applied between and within proteomes, and the result was used as input in Orthofinder to compute gene clusters. The resulting clusters of genes are called homology groups. The default parameters of Orthofinder were used. Note that a new version of Orthofinder, Orthofinder2 (Emms and Kelly 2019), was released after the completion of the present study. The results presented in this study were obtained using Orthofinder1. However, the pipeline provided to reproduce the analysis on other data sets has been updated to include Orthofinder2.

### Step 4: Preliminary Species Tree Construction Using Single-Copy Homology Groups

Single-copy homology groups were selected from the homology groups computed in Step 3. Single-copy homology groups are gene clusters containing exactly one gene from each genome. Such homology groups are considered as putative orthology groups that have evolved from a common ancestral gene without any gene duplication events. Thus, they can be used to infer a preliminary species tree using an alignment-based phylogeny estimation method. Due to HGT events, however, they may contain PTGs that should be removed before using the groups for estimating the species tree. Still, PTGs were removed from single-copy homology groups, and the remaining sequences in each group were aligned using the multiple sequence alignment software MAFFT (Katoh et al. 2002). The resulting alignments were concatenated, and the concatenated multiple alignment was used as input to the phylogeny construction method RAxML (Stamatakis 2014) to compute an initial phylogenetic tree with the set of *Corynebacteriales* genomes. The default parameters of MAFFT were used. RAxML was used with the following parameters: raxml -s alignmentfile -p 123456 -m PROTGAMMAAUTO -b 123456 -N 100 –o Nostoc_punctiforme –asc-corr lewis. The tree was rooted using the genome of *Nostoc punctiforme*—(GenBank ID: NC 010628), a symbiotic nitrogen-fixing cyanobacteria—as outgroup.

### Step 5: Gene Tree Construction and Discarding of Confirmed Transferred Genes

Among the homology groups computed at Step 3, those that contained at most one gene per genome were extracted. The set of homology groups was restricted to this set because the gene tree-based methods for species tree estimation require that gene trees contain at most one gene per genome (Mirarab and Warnow 2015; Vachaspati and Warnow 2015). For each of the 9,161 homology groups selected, a gene tree was built using the sequence alignment tool MAFFT (Katoh et al. 2002), and the phylogeny inference tool FastTree (Price et al. 2010). FastTree is a ML method that only implements partially the ML approach. It was shown to be more accurate and faster than other ML approaches for applications on large data sets. FastTree was chosen for gene tree construction because of its effectiveness in computing trees on large data sets. The default parameters of FastTree were used. The gene trees were then rooted with homologous CDSs from *N. punctiforme*, as in Step 4. A total of 631 homology groups without any homolog in *N. punctiforme* were discarded, leaving 8,530 gene trees for the analysis. Each gene tree was compared with the preliminary species tree built in Step 4 in order to double-check the classification of PTGs detected in Step 2 and correct false positives. The comparison method is as follows (see supplementary fig. S7, Supplementary Material online, for an illustration). Given any maximum complete subtree T1 of a gene tree G such that the leaves of T1 were all PTGs, we considered T2, the sibling subtree of T1 in G. The sets of species corresponding to the genes at leaves of T1 and T2 are denoted SA1 and SA2, respectively. The PTGs in T1 were reclassified as vertically inherited genes if the lowest common ancestor (lca) node of SA1 and the lca node of SA2 in the species tree S were the same node or sibling nodes. The rationale is that if T1 is the result of a HGT event from a donor branch (a, b) to an acceptor branch (a′, b′) of the species tree such that b and b′ are not sibling nodes, then the lca node of SA1 should be the node b′ and the lca node of SA2 should be the node b. Thus, in the case where lca(SA1) and lca(SA2) are the same node or sibling nodes, the hypothesis that T1 is the result of a HGT event can be discarded. The PTGs that were not reclassified were confirmed as transferred genes and removed from the homology groups and the corresponding gene trees.

### Step 6: Species Tree Construction Using Gene Tree-Based Methods

The gene trees obtained at the end of Step 5 were categorized into five collections of trees according to the maximum proportion of missing genomes in the gene trees: 20%, 40%, 50%, 60%, or 80% of missing genomes. Using each collection of trees, two species trees were constructed using the gene tree-based summary methods ASTRID (Vachaspati and Warnow 2015) and ASTRAL (Mirarab and Warnow 2015). ASTRID and ASTRAL were used in order to account for remaining horizontally transferred genes that were not detected in Step 2, either because they were missed by the GI detection method, or because they are not located in GIs. The default parameters of ASTRID and ASTRAL were used. Subsequently, each set of five trees estimated using the same gene tree-based method (ASTRID or ASTRAL) was reduced to a single consensus tree following the majority-rule consensus algorithm in CONSENSE (Felsenstein 1993). Lastly, the ASTRID consensus tree, the ASTRAL consensus tree, and the RAxML preliminary species tree from Step 4 were reduced to single overall consensus tree.

## Results

### A New Gene Tree-Based Method Applied to Estimate *Corynebacteriales* Phylogeny

We present a gene tree-based method that includes the detection and correction of putative horizontally transferred genes to estimate bacterial phylogenies using complete genome sequences (for an overview of the method, see fig. 1; for a detailed description of the six steps, see the Materials and Methods section).

The phylogeny of *Corynebacteriales* was estimated using 360 records from NCBI Reference Sequence Database, release 81 (Step 1). The 360 genomes cover 101 species and 11 genera of *Corynebacteriales*, as presented in table 1 and additional files A1 and A2.

**Table 1:** Input Data Set for *Corynebacteriales* Phylogenetic Tree Estimation

Genus	Number of Species	Number of Genomes
*Lawsonella*	1	2
*Hoyosella*	1	1
*Rhodococcus*	7	22
*Mycobacterium*	32	169
*Dietzia*	1	1
*Tsukamurella*	1	1
*Corynebacterium*	46	150
*Brevibacterium*	1	2
*Nocardia*	6	6
*Gordonia*	4	5
Total	101	360

Using parametric methods for HGT detection, 168,724 PTGs located into 2,874 GIs were detected (Step 2). Additional file A3 presents the number of GIs and PTGs detected per genome.

The gene clustering step resulted in the clustering of 1,356,782 genes (99.2% of genes) into 17,821 nonsingleton homology groups (Step 3). Additional file A4 presents the details on the composition of the homology groups.

The homology groups containing exactly one gene from each of the 360 genomes were considered as putative orthology groups. After the PTGs were removed from these groups, they were used to build a preliminary species tree using the RAxML ML phylogenetic method (Stamatakis 2014) (Step 4). Supplementary table S1, Supplementary Material online, presents the 13 putative orthology groups used in this step. Supplementary figure S1, Supplementary Material online, shows the preliminary species tree.

The preliminary species tree was then used to check the PTGs in the homology groups using a phylogenetic approach that consists in comparing the gene trees of homology groups with the species tree. Using this approach, 13,966 PTGs (8.29% of PTGs) were reclassified as vertically inherited genes (Step 5).

The gene trees corresponding to homology groups with, at most, one gene per genome were clustered into five collections of trees according to the maximum proportion of genomes without any gene in the homology group: 20%, 40%, 50%, 60%, and 80%. For instance, gene trees in which 45% of genomes did not have a gene were included in the 50%, 60%, or 80% groups. Supplementary table S2, Supplementary Material online, provides the number of trees in the five resulting collections.

Two gene tree-based phylogenetic methods—ASTRID (Vachaspati and Warnow 2015) and ASTRAL II (Mirarab and Warnow 2015)—were applied to the 5 collections to generate 10 phylogenies on the 360 input genomes. ASTRID and ASTRAL are methods motivated by, and statistically consistent with, the multispecies coalescent model such that there is free recombination between, but not within, loci. The use of ASTRID and ASTRAL is motivated by the presence of horizontally transferred genes in the data, not detected in Step 2. The trees obtained using ASTRID and ASTRAL were rooted with the outgroup method by including a homologous CDS from the species *N. punctiforme*. In order to evaluate the similarity between the estimated phylogenies, the percentage of conserved clades between each pair of trees was computed (see table 2). The average pairwise similarity between ASTRID trees is 75.89% with values ranging from 67.22% to 87.78%. ASTRAL trees display a higher average pairwise similarity of 80.14% with values ranging from 74.17% to 89.17%. The average pairwise similarity between ASTRID trees and ASTRAL trees is 67.89% with values ranging from 65.56% to 73.89%.

**Table 2:** Square Matrix of Percentage of Conserved Clades between Phylogenies Estimated Using RAxML (Step 4), and ASTRID and ASTRAL (Step 5)

	(1)	(2)	(3)	(4)	(5)	(6)	(7)	(8)	(9)	(10)	(11)
RAxML (1)	100	82.65	82.14	83.16	82.65	82.14	81.12	81.12	80.10	81.12	81.63
ASTRID20 (2)		100	87.78	74.17	74.44	67.22	68.61	70.28	66.67	66.67	66.11
ASTRID40 (3)		—	100	76.11	75.56	68.61	67.5	69.17	67.22	67.78	66.67
ASTRID50 (4)		—	—	100	83.61	72.5	65.56	67.22	68.61	69.17	68.06
ASTRID60 (5)		—	—	—	100	78.89	66.11	67.22	68.06	70.56	70.28
ASTRID80 (6)		—	—	—	—	100	65.83	65.83	66.39	68.89	73.89
ASTRAL20 (7)		—	—	—	—	—	100	87.22	77.78	75.0	74.17
ASTRAL40 (8)		—	—	—	—	—	—	100	78.89	77.78	75.83
ASTRAL50 (9)		—	—	—	—	—	—	—	100	89.17	80.0
ASTRAL60 (10)		—	—	—	—	—	—	—	—	100	85.56
ASTRAL80 (11)		—	—	—	—	—	—	—	—	—	100

Considering the high similarity between the five phylogenies estimated using each of the two methods, the trees estimated with ASTRID, on one side, and with ASTRAL, on the other, were reduced to two consensus trees with the CONSENSE majority-rule consensus tool (Felsenstein 1993). Supplementary table S3, Supplementary Material online, presents the similarity between the two resulting consensus trees and the ten initially estimated phylogenies. The two consensus trees for ASTRID and ASTRAL have a high percentage of conserved clades (78.27%), and 82.14% and 80.61%, respectively, of conserved clades with the preliminary species tree from Step 4 obtained with RAxML. Therefore, a final reduction of the three phylogenies—ASTRID consensus, ASTRAL consensus and RAxML—to a single consensus tree (referred to as overall consensus) was made. Supplementary figure S2, Supplementary Material online, depicts the four trees viewed at the genus level. All 14 trees generated in this research are available on the iTOL webserver (Letunic and Bork 2019), at https://itol.embl.de/shared/cobius_udes (last accessed April 2, 2020).

**Fig. 2.**
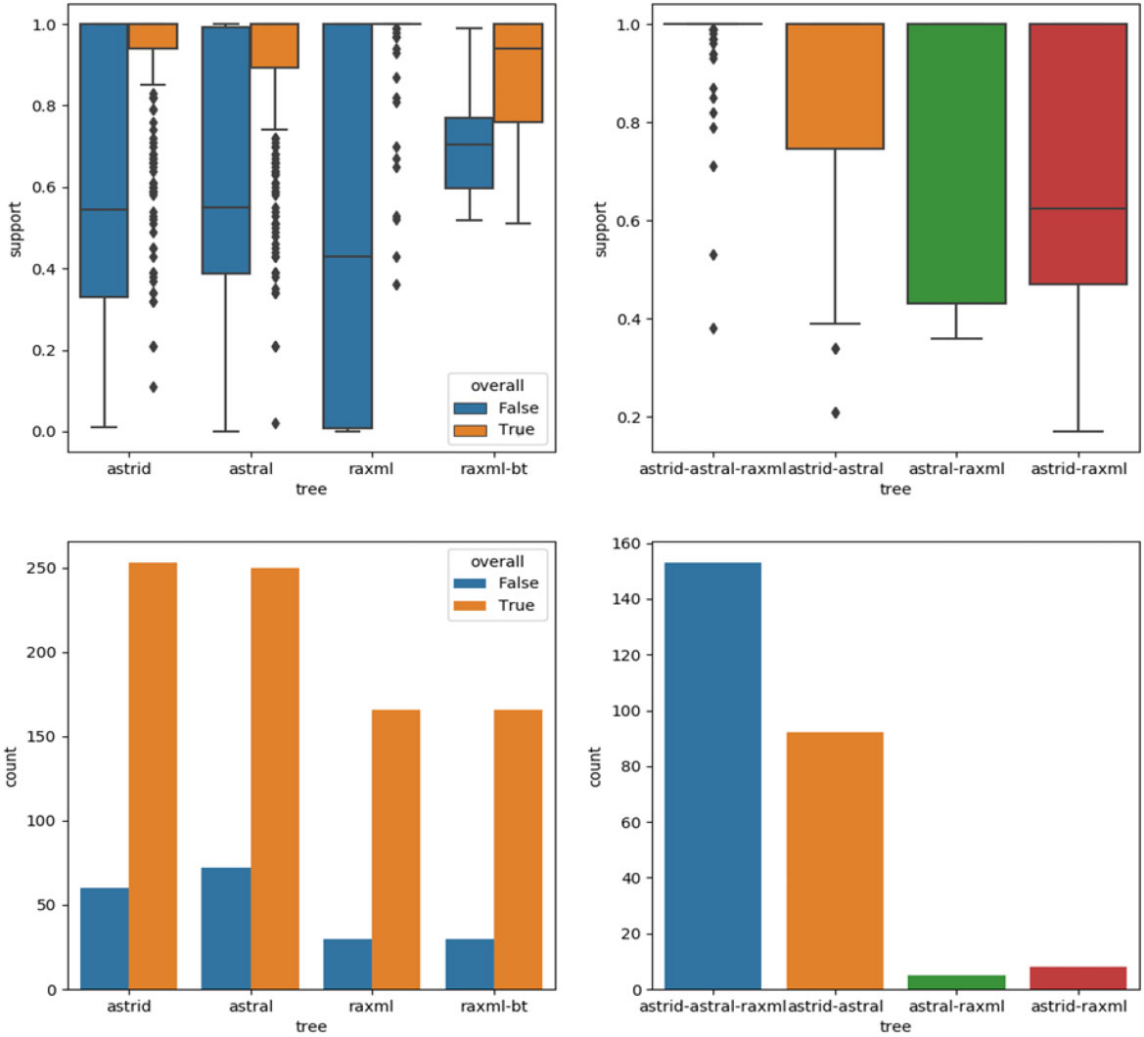
—Support values of internal branches of the ASTRID, ASTRAL, RAxML, and Overall consensus trees. Bottom-left: Numbers of branches in the ASTRID, ASTRAL, and RAxML trees conserved in the overall consensus tree and numbers of branches not conserved. Top-left: Boxplots of the quartet support values of branches in each group and the bootstrap support values for the RAxML tree. Bottom-right: Numbers of branches in the overall consensus tree present in the ASTRID, ASTRAL, and RAxML trees, or in only two of the three trees. Top-right: Boxplots of the quartet support values of branches in each group.

ASTRAL was used to compute the quartet supports of branches in the four trees. The quartet support of a branch is computed using the percentage of quartets in input gene trees that agree or disagree with the branch (Sayyari and Mirarab 2016). For each of the ASTRID, ASTRAL, and RAxML trees, the internal branches (nontrivial clades) were divided into two groups: those conserved in the overall consensus tree, and those that were not conserved. Figure 2 (bottom-left) presents the number of branches in each of the six groups, and figure 2 (top-left) presents boxplots of the quartet supports of branches in the six groups. For RAxML, the boxplots of the bootstrap supports of branches are also depicted. We observe that the clades from ASTRID, ASTRAL, and RAxML included in the overall consensus show high quartet support values, whereas the clades not included in the overall consensus show low quartet support values. The same observation holds for the RAxML bootstrap values. This means that the overall consensus tree is effective at retaining the clades of the three input trees which present the highest support values.

Table 3 presents the similarity measures between the four trees. The overall consensus has, respectively, 98.06%, 96.90%, and 84.69% of conserved clades with the ASTRID, ASTRAL, and RAxML trees. The internal branches of the overall consensus tree were divided into four groups depending on their presence in all three input trees (ASTRID, ASTRAL, and RAxML) or in only two of the input trees. Figure 2 (bottom-right) presents the number of internal branches in each of the four groups, and figure 2 (top-right) presents boxplots of the quartet supports of branches in the four groups. The branches present in all three input trees (ASTRID–ASTRAL–RAxML) constitute the largest group with the highest support values, followed by the ASTRID–ASTRAL branches, a few ASTRID–RAxML branches, and finally a few ASTRAL–RAxML branches. This means that the largest contribution comes from the consensus between the three trees or between ASTRID and ASTRAL trees, and further branches are added thanks to the consensus with the RAxML tree.

**Table 3:** Square Matrix of the Percentage of Conserved Clades between the Consensus Trees and the Preliminary RAxML Tree

	(1)	(2)	(3)	(4)
ASTRID consensus (1)	100	78.27	82.14	98.06
ASTRAL consensus (2)		100	80.61	96.90
RAxML (3)		—	100	84.69
Overall consensus (4)		—	—	100

### Analysis at the Genus Level

**Fig. 3.**
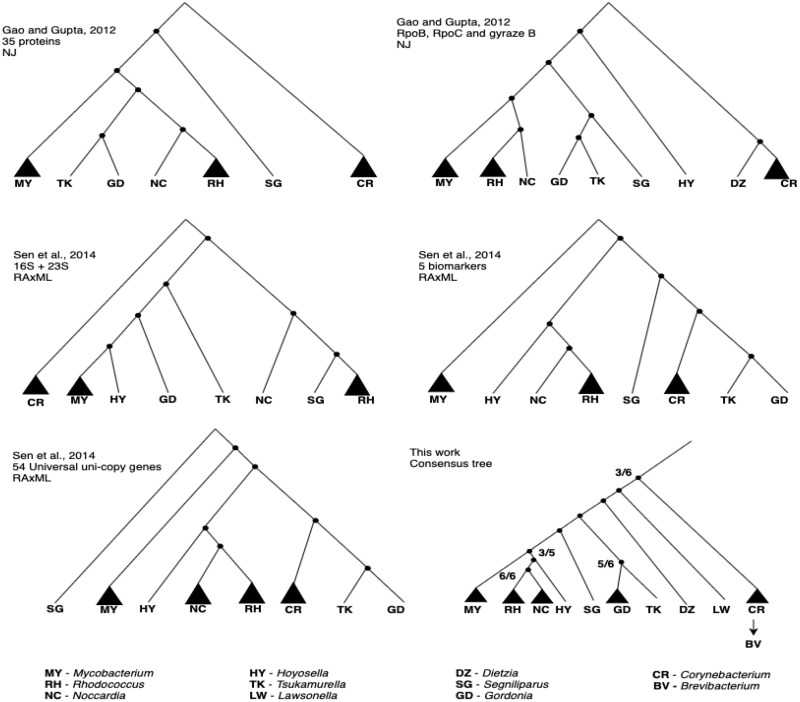
—Illustration at the genus level of the overall consensus phylogeny reconstructed in our work, two phylogenies reconstructed in Gao and Gupta (2012), and three phylogenies reconstructed in Sen et al. (2014). For each tree, the genera for which the data set contains more than one species are represented as triangles. Note that the sets of genera differ between trees. The ratio of trees displaying the clade is indicated for each conserved clade.

All the trees estimated in our study place the genus *Brevibacterium* (BV) within *Corynebacterium* (CR), which supports reclassifying *Brevibacterium* as *Corynebacterium*, as proposed in a recent study (Yang and Yang 2017). The *Corynebacteriales* phylogeny is still under debate. Past studies have reported various topologies for the phylogeny of this order. Gao and Gupta (2012) used the sequence alignments of 35 proteins with neighbor-joining methods to estimate the phylogeny of *Actinobacteria* that includes *Corynebacteriales*. Sen et al. (2014) used 54 protein sequences aligned with RAxML to infer a phylogeny on 100 actinobacterial strains. They also reported a second phylogeny on the 100 actinobacterial strains obtained by applying RAxML to the alignment of 5 conserved genes identified with a multilocus sequence analysis. We compared the overall consensus tree obtained in our study with the *Corynebacteriales* phylogenies from Gao and Gupta (2012) and Sen et al. (2014). Figure 3 depicts the compared phylogenies at the genus level. A strong consensus can be seen between the trees for the clade grouping *Nocardia* and *Rhodococcus* (6/6) and the clade regrouping *Gordonia* and *Tsukamurella* (5/6). We also observed a majority-rule consensus for a clade grouping *Nocardia*, *Rhodococcus*, and *Hoyosella* (3/5), and for placing *Corynebacterium* as the outgroup (3/6).

### Systematic Analysis of the Phylogeny Reported inside Genera

We analyzed the phylogenies for each of the 5 genera with more than 1 species in the data set: *Corynebacterium* (46 species), *Mycobacterium* (32 species), *Rhodoccoccus* (7 species), *Nocardia* (6 species), and *Gordonia* (4 species). In all the trees estimated in our study, the species of the same genus were grouped into monophyletic groups. We extracted the complete subtree corresponding to each genus in the overall consensus tree.

#### Corynebacterium

**Fig. 4.**
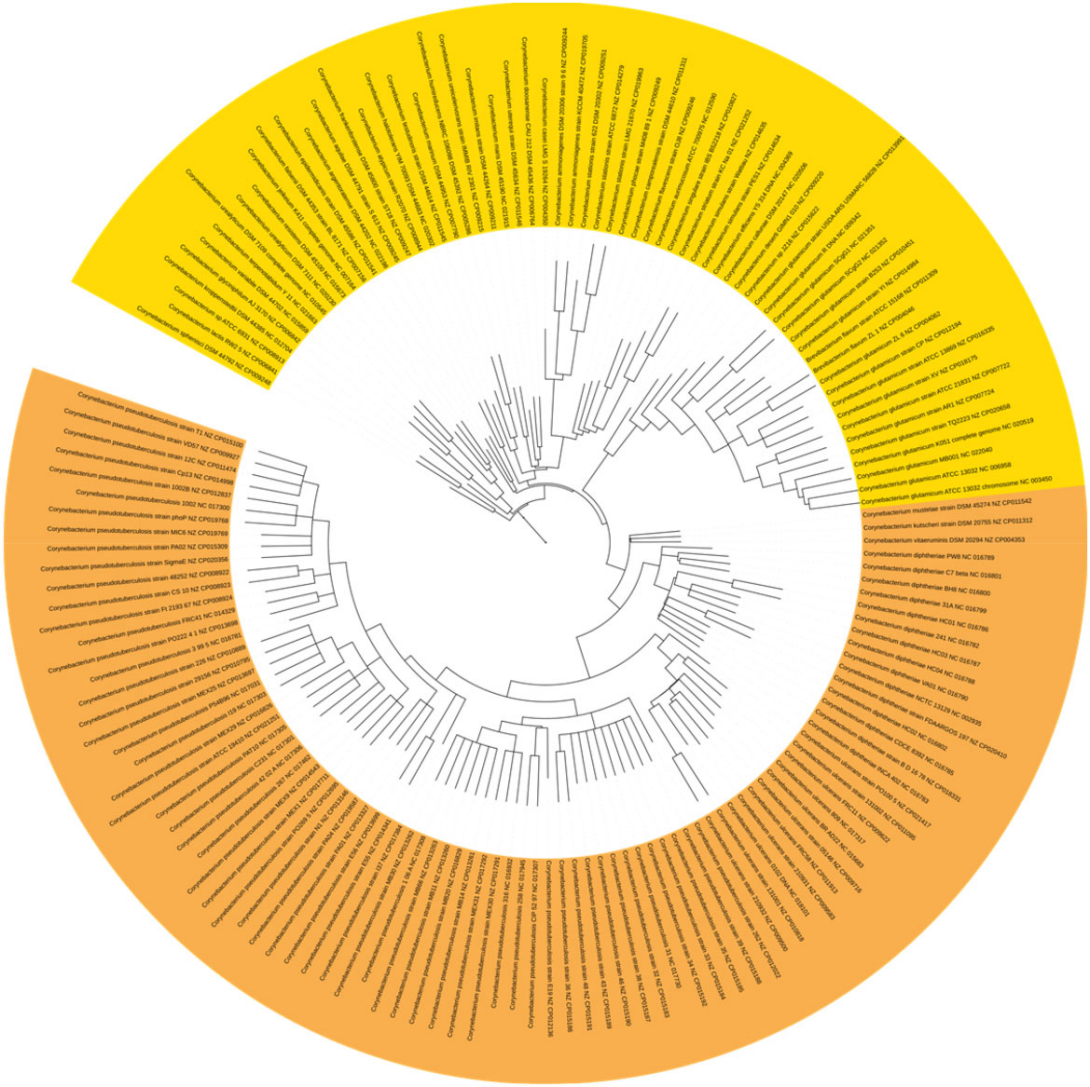
—Partition of *Corynebacterium* into two categories: nonpathogenic in yellow and pathogenic in orange. Detailed method used to estimate this phylogeny (overall consensus tree: RAxML + ASTRID consensus + ASTRAL consensus).

The genus *Corynebacterium* comprises a variety of bacterial species that includes potential pathogens for human and animals, as well as pathogens for normal microbiota (Von Graevenitz and Bernard 2006). Most of the mechanisms underlying diseases caused by these species are still unclear; a few phylogenies of the genus have been reconstructed (Pascual et al. 1995; Baek et al. 2018; Dangel et al. 2019). In all the trees estimated in our study, we observed a division into two categories: nonpathogenic genomes and pathogenic genomes forming a monophyletic group (fig. 4). We noted a single exception: the classification of *Corynebacterium jeikeium* among nonpathogenic genomes. *Corynebacterium jeikeium* is a pathogen isolated from immunosuppressive patients highly exposed to antibiotic treatments (Tauch et al. 2005). The positioning of this pathogen species among nonpathogens is surprising and might be related to HGT for the acquisition of antibiotic-resistance genes.

In fact, as it was only isolated from immunosuppressive patients, this is a robust biological clue suggesting that this species does not act as a pathogen in healthy organisms (in this case, humans). Therefore, it is not a primary pathogen, which would corroborate our findings. More detailed studies are needed to refine this assumption. In the nonpathogenic group, *Corynebacterium glutamicum* is the most thoroughly studied species due to its biotechnological applications in producing amino acids such as l-arginine, l-histidine, l-carnitine, l-lysine, and l-valine (Keilhauer et al. 1993). In all the trees estimated in our study, the genomes of *Brevibacterium flavum* strain ATCC 15168 (RefSeq. CP011309) and *B. flavum* ZL 1 (RefSeq. CP004046) always appear in the same clade of the genomes of *C. glutamicum* (supplementary fig. S3, Supplementary Material online). This classification was recently proposed in the literature (Yang and Yang 2017).

In the pathogenic group, we observe a clustering of *Corynebacterium diphteriae* and *Corynebacterium ulcerans. Corynebacterium diphteriae* is the etiological agent of diphtheria in humans, an infectious disease caused by the exotoxin produced by this pathogen (Cerdeno-Tarraga et al. 2003). *Corynebacterium ulcerans* is primarily reported for causing mastitis in cattle and humans due to the consumption of raw milk or unpasteurized dairy products in rural populations (Hommez et al. 1999). In the literature, *C. ulcerans* has been closely related to *C. diphtheriae*, which produces a toxin causing symptoms similar to those caused by *C. ulcerans* (Riegel et al. 1995).

**Fig. 5.**
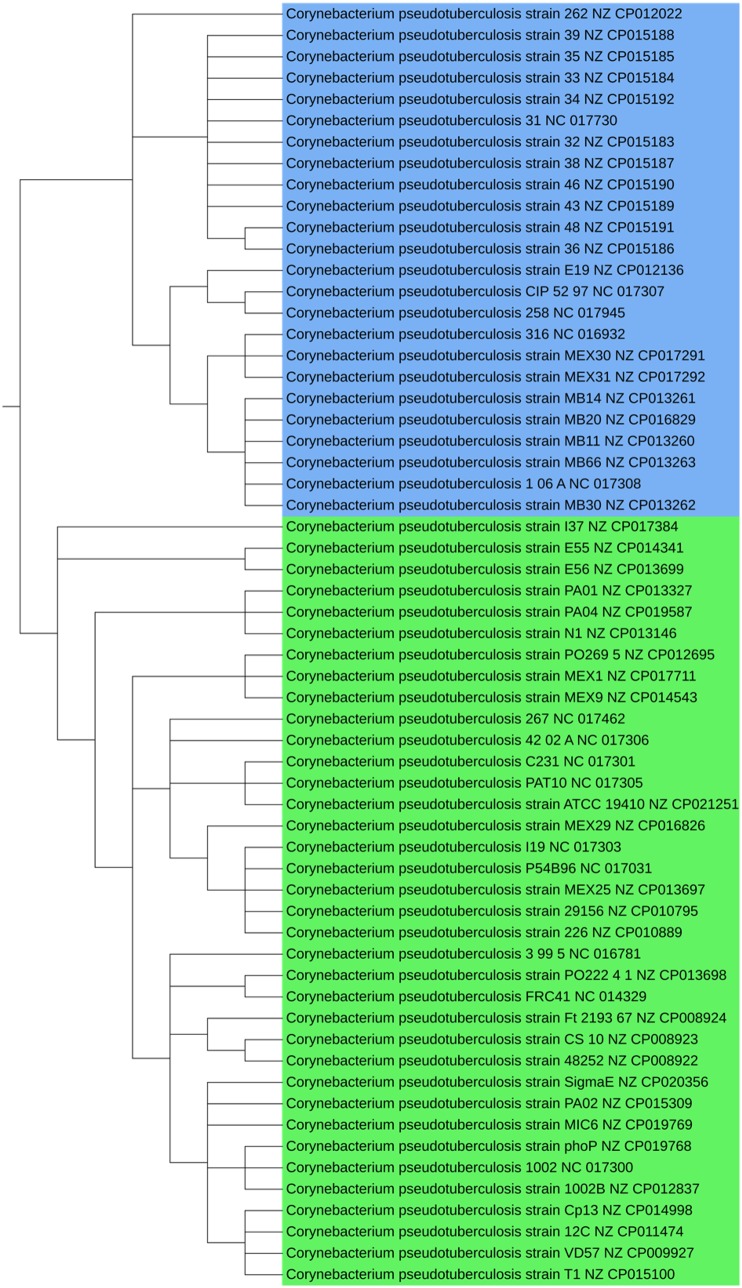
—Partition of *C. pseudotuberculosis* into two biovars. Biovars equi and ovis are shown in green and blue, respectively. Detailed method used to estimate this phylogeny (overall consensus tree: RAxML + ASTRID consensus+ ASTRAL consensus).

We also detected the recently proposed anagenesis of *Corynebacterium pseudotuberculosis* (Oliveira et al. 2016). In this model, two biovars are described: equi and ovis. They mainly differ by the presence of the nitrate reductase enzyme present in biovar equi, which results in 1% of the nucleotide differences between biovars (Soares et al. 2013) (fig. 5). *Corynebacterium pseudotuberculosis* is the etiological agent of caseous lymphadenitis (CLA), a highly prevalent chronic disease affecting sheep and goats. It is difficult to control and causes significant economic losses to farmers (Baird and Fontaine 2007). Human infections caused by *C. pseudotuberculosis* are rare, but it has been reported as the agent of necrotizing lymphadenitis in human (Mills et al. 1997). Lastly, we observed that our phylogeny for *Corynebacterium* species is consistent with the phylogeny proposed by Gao and Gupta (2012).

#### Mycobacterium

**Fig. 6.**
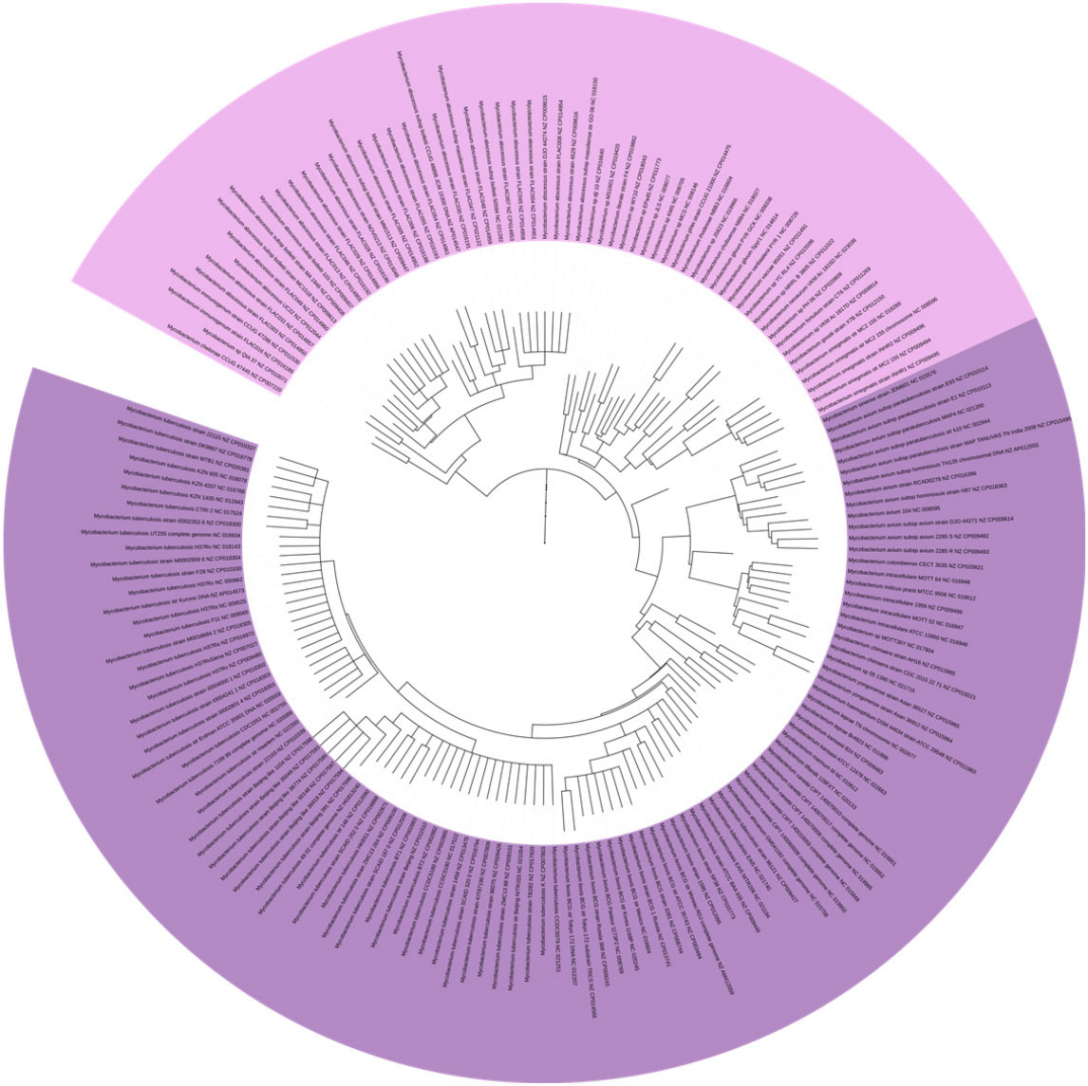
—Partition of *Mycobacterium* into two categories: slow growers in purple and fast growers in pale pink. Detailed method used to estimate this phylogeny (overall consensus tree: RAxML + ASTRID consensus+ ASTRAL consensus).

The genus *Mycobacterium* comprises one the most dangerous human pathogens—*Mycobacterium tuberculosis*—which causes tuberculosis (Gagneux 2018; Koch and Mizrahi 2018). This genus also comprises others important animal pathogens such as *Mycobacterium leprae*, *Mycobacterium bovis*, and *Mycobacterium avium* (Frothingham and Wilson 1993). The taxonomy of *Mycobacterium* solely relies in two categories: slow growers and fast growers. This poorly detailed taxonomy is due to the lack of descriptive features for taxonomic classification. A more detailed classification would help with global monitoring of disease outbreaks caused by species of this genus (Rogall et al. 1990; Stahl and Urbance 1990). All the estimated trees display a division into the 2 categories: 61 genomes of slow growers forming a monophyletic group, and 108 genomes of fast growers forming another monophyletic group (fig. 6).

#### Rhodococcus

The *Rhodococcus* species are used as versatile genetic tools in the biotechnological industry because of their capacity for remediation, biotransformation and biocatalysis, biodegradation of diverse metabolic compounds, adaptation and tolerance to solvents, and interactions with metals (Sangal et al. 2019). *Rhodococcus* species are distributed in soil, water, and marine sediments (Larkin et al. 2005). Some of them are also pathogens for humans, animals, and plants (Prescott 1991). Although new *Rhodococcus* genomes are still being sequenced because of their important biotechnological applications, the current phylogenies of *Rhodococcus* are only estimated for closely related species using few biomarkers (Anastasi et al. 2016; Duquesne et al. 2017). We report a phylogeny of 22 *Rhodococcus* genomes corresponding to 6 species and 11 unclassified genomes, divided into 6 clusters (supplementary fig. S4, Supplementary Material online). The lack of estimated phylogenies for *Rhodococcus* at the species level in the literature makes it hard to conduct a proper comparison with past studies. We however observed partial agreement of the estimated phylogeny for the six *Rhodococcus* species with the phylogeny from (Anastasi et al. 2016): (*R. fascians*,(*R. pyridinovorans*, (*R. erythropolis*,(*R. opacus*, *R. jostii*)))).

#### Nocardia

*Nocardia* species are a complex group of organisms that cause serious human infections, especially in immunocompromised patients. Like *Rhodococcus* and the other genera of *Cornynebacteriales*, the taxonomy and phylogeny of *Nocardia* species are subject to open debate (Conville et al. 2018). The data set contains six complete genomes of *Nocardia* corresponding to six species. All the estimated trees display the same phylogeny for the six species (supplementary fig. S5, Supplementary Material online). In an estimation of the phylogeny of *Nocardia* species, Conville et al. (2018) described the complex history behind the taxonomy of the genus by reconstructing a phylogenetic tree using the 16S rRNA gene of 59 genomes of *Nocardia*. The intersection between their data set and our data set consists of only four species. The estimated phylogeny for these four species in (Conville et al. 2018) is ((*N. farcinica*, *N. brasilensis*), (*N. cyriacigeorgica*, *N. nova*)), which differs from the phylogeny estimated in this report.

#### Gordonia

*Gordonia* species have attracted interest from the biotechnological industry in recent years because of their ability to degrade environmental pollutants as well as natural polymers and compounds, making them potentially useful for environmental and industrial biotechnology (Arenskötter et al. 2004). Some species of *Gordonia* are reported to cause infections in humans (Ramanan et al. 2013; Sowani et al. 2017). Previous phylogenies of *Gordonia* were estimated with 16S rRNA genes, and the phylogeny is still under debate (Blaschke et al. 2007). Kang et al. (2009) studied the phylogeny of 23 species using *gyrB*, *secA1*, and 16S rRNA genes. In our study, we collected five *Gordonia* genomes, corresponding to three species and two unclassified genomes. All the estimated trees display the same phylogeny for the five genomes (supplementary fig. S6, Supplementary Material online). The induced phylogeny for the three species included in our data set—*G. polyisoprenivorans*, *G. bronchialis*, and *G. terrae*—agrees with the induced phylogeny from Kang et al. (2009).

## Discussion

As new bacterial genomes are still being sequenced, one of the major problems lies with identifying the main bacteria groups and recovering the phylogenetic relationships between these groups (Larson 1998). Current modern molecular-biology techniques are still being redesigned to identify new species because the classical approaches based on sequence analysis are inefficient for discrimination (Glaeser and Kämpfer 2015). Furthermore, characterizing the differences between closely related species remains challenging (Christensen and Olsen 2018). Sequence-based phylogenies have been an active research field since the beginning of the 2000s. They grounded the current knowledge about the diversity of organisms on the Earth. Estimating bacterial phylogenies is not, however, a trivial problem. This is mainly because bacterial genomes are highly affected by the swapping of genetic material between genomes via HGT processes (Soucy et al. 2015). Through this mechanism, bacterial genomes acquire and spread genes that confer adaptive advantages, such as antibiotic-resistance genes leading to the rise of multidrug-resistant bacteria (Van Duin and Paterson 2016). Thus, accounting for horizontally transferred genes is necessary to accurately estimate bacterial phylogenies when using sequence-based phylogenetic methods. Nonetheless, current sequence-based phylogenetic methods do not include a step back to audit the data sets in order to identify and remove transferred genes. An alternative to identifying and removing transferred genes before estimating phylogenies is to infer ancestral recombination graphs that record of all coalescence and recombination events in the evolution of a set of homologous sequences (D O’Fallon 2013; Rasmussen et al. 2014). However, existing methods for ancestral recombination graphs inference are computationally intensive and limited to small numbers of sequences. Herein, the phylogeny of *Corynebacteriales* was estimated while accounting for HGTs, by detecting and removing a part of the HGT located in GIs using a parametric GI detection method, and by relying on phylogenetic reconstruction methods which are consistent the multispecies coalescent model with recombination within loci. The result is a species tree that displays all the genera as monophyletic clades. The estimated trees display several phylogenetic relationships proposed by previous studies: 1) the classification of *B. flavum* inside *C. glutamicum* (Yang and Yang 2017), 2) the monophyletic group composed of pathogens *C. ulcerans* and *C. diphteriae*, 3) the biovar speciation inside *C. pseudotuberculosis*, and 4) the division between slow growers and fast growers in *Mycobacterium*. Finally, it is important to recall that the phylogenomics method devised in this article presents the same limit as most phylogenetics and comparative genomics methods which reduce biological processes such as HGT to patterns, and thus investigate patterns (Nelson 1970). One should always remember that phylogenetics methods are consistent under the hypothesis that there is a one-to-one correspondence between the target biological processes and the patterns investigated.

## Data Availability 

All information to retrieve the data and the scripts used for the analysis are available on the CoBIUS lab GitHub (https://github.com/UdeS-CoBIUS/EXECT).

## Supplementary Material

evaa058_Supplementary_Data
