# Supplementary material for: Reconstructing the Phylogeny of Corynebacteriales while Accounting for Horizontal Gene Transfer
Source: Genome Biol Evol. 2020 Apr 2;12(4):381–95. doi: 10.1093/gbe/evaa058 (PMC7186787; doi:10.1093/gbe/evaa058)
Supplement: evaa058_Supplementary_Data [file evaa058_supplementary_data.zip › Additional_files_A1-A4.pdf]

| Additional file A1 - Number of genomes per species | | |
|---|---|---|
| Genus | Species | Number of genomes |
| *Lawsonella* | *clevelandensis* | 2 |
| *Hoyosella* | *subflava* | 1 |
| *Rhodococcus* | *erythropolis* | 4 |
| *Rhodococcus* | *fascians* | 1 |
| *Rhodococcus* | *jostii* | 1 |
| *Rhodococcus* | *pyridinivorans* | 1 |
| *Rhodococcus* | *aetherivorans* | 1 |
| *Rhodococcus* | *sp.* | 11 |
| *Rhodococcus* | *opacus* | 3 |
| *Mycobacterium* | *vaccae* | 1 |
| *Mycobacterium* | *tuberculosis* | 57 |
| *Mycobacterium* | *kansasii* | 2 |
| *Mycobacterium* | *avium* | 12 |
| *Mycobacterium* | *colombiense* | 1 |
| *Mycobacterium* | *intracellulare* | 3 |
| *Mycobacterium* | *goodii* | 1 |
| *Mycobacterium* | *sinense* | 1 |
| *Mycobacterium* | *leprae* | 2 |
| *Mycobacterium* | *abscessus* | 29 |
| *Mycobacterium* | *marinum* | 2 |
| *Mycobacterium* | *liflandii* | 1 |
| *Mycobacterium* | *chimaera* | 2 |
| *Mycobacterium* | *immunogenum* | 2 |
| *Mycobacterium* | *paraintracellulare* | 1 |
| *Mycobacterium* | *bovis* | 13 |
| *Mycobacterium* | *chubuense* | 1 |
| *Mycobacterium* | *africanum* | 1 |
| *Mycobacterium* | *yongonense* | 3 |
| *Mycobacterium* | *indicus* | 1 |
| *Mycobacterium* | *rhodesiae* | 1 |
| *Mycobacterium* | *smegmatis* | 5 |
| *Mycobacterium* | *haemophilum* | 1 |
| *Mycobacterium* | *sp.* | 15 |
| *Mycobacterium* | *phlei* | 1 |
| *Mycobacterium* | *vanbaalenii* | 1 |
| *Mycobacterium* | *canettii* | 5 |
| *Mycobacterium* | *gilvum* | 2 |
| *Mycobacterium* | *fortuitum* | 1 |
| *Mycobacterium* | *neoaurum* | 1 |
| *Mycobacterium* | *litorale* | 1 |
| *Mycobacterium* | *chelonae* | 1 |

| | | |
|---|---|---|
| *Dietzia* | *timorensis* | 1 |
| *Tsukamurella* | *paurometabola* | 1 |
| *Corynebacterium* | *atypicum* | 1 |
| *Corynebacterium* | *doosanense* | 1 |
| *Corynebacterium* | *imitans* | 1 |
| *Corynebacterium* | *ulcerans* | 11 |
| *Corynebacterium* | *callunae* | 1 |
| *Corynebacterium* | *terpenotabidum* | 1 |
| *Corynebacterium* | *testudinoris* | 1 |
| *Corynebacterium* | *uterequi* | 1 |
| *Corynebacterium* | *epidermidicanis* | 1 |
| *Corynebacterium* | *ammoniagenes* | 2 |
| *Corynebacterium* | *kroppenstedtii* | 1 |
| *Corynebacterium* | *urealyticum* | 2 |
| *Corynebacterium* | *camporealensis* | 1 |
| *Corynebacterium* | *kutscheri* | 1 |
| *Corynebacterium* | *simulans* | 2 |
| *Corynebacterium* | *vitaeruminis* | 1 |
| *Corynebacterium* | *phocae* | 1 |
| *Corynebacterium* | *resistens* | 1 |
| *Corynebacterium* | *aquilae* | 1 |
| *Corynebacterium* | *efficiens* | 1 |
| *Corynebacterium* | *argentoratense* | 1 |
| *Corynebacterium* | *humireducens* | 1 |
| *Corynebacterium* | *glyciniphilum* | 1 |
| *Corynebacterium* | *crudilactis* | 1 |
| *Corynebacterium* | *diphtheriae* | 15 |
| *Corynebacterium* | *marinum* | 2 |
| *Corynebacterium* | *ureicelerivorans* | 1 |
| *Corynebacterium* | *lactis* | 1 |
| *Corynebacterium* | *deserti* | 1 |
| *Corynebacterium* | *casei* | 1 |
| *Corynebacterium* | *singulare* | 1 |
| *Corynebacterium* | *halotolerans* | 1 |
| *Corynebacterium* | *glutamicum* | 17 |
| *Corynebacterium* | *maris* | 1 |
| *Corynebacterium* | *frankenforstense* | 1 |
| *Corynebacterium* | *sphenisci* | 1 |
| *Corynebacterium* | *pseudotuberculosis* | 60 |
| *Corynebacterium* | *aurimucosum* | 1 |
| *Corynebacterium* | *striatum* | 1 |
| *Corynebacterium* | *flavescens* | 1 |
| *Corynebacterium* | *stationis* | 3 |
| *Corynebacterium* | *sp.* | 2 |
| *Corynebacterium* | *falsenii* | 1 |
| *Corynebacterium* | *variabile* | 1 |
| *Corynebacterium* | *jeikeium* | 1 |
| *Corynebacterium* | *mustelae* | 1 |
| *[Brevibacterium]* | *flavum* | 2 |

| | | |
|---|---|---|
| *Nocardia* | *farcinica* | 1 |
| *Nocardia* | *seriolae* | 1 |
| *Nocardia* | *soli* | 1 |
| *Nocardia* | *nova* | 1 |
| *Nocardia* | *cyriacigeorgica* | 1 |
| *Nocardia* | *brasiliensis* | 1 |
| *Gordonia* | *polyisoprenivorans* | 1 |
| *Gordonia* | *bronchialis* | 1 |
| *Gordonia* | *terrae* | 1 |
| *Gordonia* | *sp.* | 2 |
| *Segniliparus* | *rotundus* | 1 |

Additional file A2 - Size and number of CDS per genome

| Genome ID | Definition | Chromossome Size (bp) | Number of CDS |
|---|---|---|---|
| NZ_AM412059 | Mycobacterium bovis BCG str. Moreau RDJ complete genome. | 4340116 | 4158 |
| NZ_CP010818 | Corynebacterium ulcerans strain 131001 | 2483321 | 2196 |
| NC_010545 | Corynebacterium urealyticum DSM 7109 complete genome. | 2369219 | 1996 |
| NC_020302 | Corynebacterium halotolerans YIM 70093 = DSM 44683 | 3135752 | 2794 |
| NZ_CP016640 | Mycobacterium sp. djl-10 | 6395946 | 6085 |
| NC_016906 | Gordonia polyisoprenivorans VH2 | 5669805 | 5014 |
| NZ_CP015529 | Rhodococcus sp. WB1 | 5924026 | 5301 |
| NC_021054 | Mycobacterium tuberculosis str. Beijing/NITR203 | 4411128 | 4244 |
| NZ_CP015961 | Dietzia timorensis strain ID05-A0528 | 3607892 | 3265 |
| NZ_CP011883 | Mycobacterium haemophilum DSM 44634 strain ATCC 29548 | 4235765 | 3976 |
| NZ_CP017014 | Rhodococcus sp. WMMA185 | 4444448 | 3949 |
| NZ_CP015773 | Mycobacterium bovis strain SP38 | 4347648 | 4165 |
| NZ_CP018301 | Mycobacterium tuberculosis strain I0002801-4 | 4376067 | 4203 |
| NZ_CP009583 | Corynebacterium ulcerans strain 210931 | 2503722 | 2238 |
| NZ_CP016188 | Mycobacterium abscessus strain FLAC006 | 4891993 | 4810 |
| NC_009525 | Mycobacterium tuberculosis H37Ra | 4419977 | 4245 |
| NZ_CP009480 | Mycobacterium tuberculosis H37Rv | 4396119 | 4232 |
| NC_002935 | Corynebacterium diphtheriae NCTC 13129 | 2488635 | 2310 |
| NC_008769 | Mycobacterium bovis BCG Pasteur 1173P2 | 4374522 | 4186 |
| NC_021663 | Corynebacterium terpenotabidum Y-11 | 2751233 | 2376 |
| NZ_CP010113 | Mycobacterium avium subsp. paratuberculosis strain E1 | 4781002 | 4504 |
| NZ_CP020410 | Corynebacterium diphtheriae strain FDAARGOS_197 | 2489000 | 2305 |
| NC_021915 | Corynebacterium maris DSM 45190 | 2787574 | 2536 |
| NZ_CP016829 | Corynebacterium pseudotuberculosis strain MB20 | 2370901 | 2128 |
| NZ_CP009245 | Corynebacterium aquilae DSM 44791 strain S-613 | 2926437 | 2397 |
| NC_023150 | Rhodococcus pyridinivorans SB3094 | 5227080 | 4748 |
| NZ_CP013262 | Corynebacterium pseudotuberculosis strain MB30 | 2364377 | 2124 |
| NZ_CP013741 | Mycobacterium bovis strain BCG-1 (Russia) | 4370705 | 4185 |
| NZ_CP020658 | Corynebacterium glutamicum strain TQ2223 | 3306634 | 3039 |
| NZ_CP005286 | Corynebacterium humireducens NBRC 106098 = DSM 45392 | 2681312 | 2526 |
| NZ_CP014279 | Corynebacterium stationis strain ATCC 6872 | 2853666 | 2585 |
| NC_016946 | Mycobacterium intracellulare ATCC 13950 | 5402402 | 5065 |
| NZ_CP014956 | Mycobacterium abscessus strain FLAC029 | 5188507 | 5151 |
| NZ_CP014635 | Corynebacterium simulans strain Wattiau | 2598702 | 2365 |
| NZ_CP009244 | Corynebacterium ammoniagenes DSM 20306 strain 9.6 | 2790185 | 2482 |
| NZ_CP009615 | Mycobacterium abscessus strain DJO-44274 | 4686331 | 4622 |
| NZ_CP010889 | Corynebacterium pseudotuberculosis strain 226 | 2337820 | 2079 |
| NC_017303 | Corynebacterium pseudotuberculosis I19 | 2337594 | 2079 |
| NC_017462 | Corynebacterium pseudotuberculosis 267 | 2337628 | 2077 |
| NC_016604 | Mycobacterium rhodesiae NBB3 | 6415739 | 6200 |
| NZ_CP014951 | Mycobacterium abscessus strain FLAC004 | 5242371 | 5281 |
| NZ_CP014955 | Mycobacterium abscessus strain FLAC013 | 5074222 | 5013 |
| NC_019965 | Mycobacterium canettii CIPT 140070008 complete genome. | 4420197 | 4203 |
| NC_017730 | Corynebacterium pseudotuberculosis 31 | 2402956 | 2159 |
| NC_020245 | Mycobacterium bovis BCG str. Korea 1168P | 4376711 | 4190 |
| NZ_CP007255 | Rhodococcus erythropolis R138 | 6236862 | 5688 |
| NC_021352 | Corynebacterium glutamicum SCgG2 | 3350619 | 3049 |
| NC_018581 | Gordonia sp. KTR9 | 5441391 | 4838 |
| NZ_CP020809 | Mycobacterium sp. PH-06 | 7595921 | 7225 |
| NZ_CP004353 | Corynebacterium vitaeruminis DSM 20294 | 2931780 | 2569 |
| NZ_CP003494 | Mycobacterium bovis BCG str. ATCC 35743 | 4334064 | 4175 |
| NC_018681 | Nocardia brasiliensis ATCC 700358 | 9436348 | 8434 |
| NZ_CP018303 | Mycobacterium tuberculosis strain I0004241-1 | 4386132 | 4207 |
| NC_014168 | Segniliparus rotundus DSM 44985 | 3157527 | 3033 |
| NZ_CP007027 | Mycobacterium tuberculosis H37RvSiena | 4410911 | 4236 |
| NC_000962 | Mycobacterium tuberculosis H37Rv | 4411532 | 3906 |
| NZ_CP009312 | Corynebacteriales bacterium X1036 | 1860551 | 1559 |
| NZ_CP008744 | Mycobacterium bovis BCG strain 3281 | 4410431 | 4217 |
| NC_008726 | Mycobacterium vanbaalenii PYR-1 | 6491865 | 6142 |
| NZ_CP016192 | Mycobacterium abscessus strain FLAC046 | 5214168 | 5180 |
| NC_002944 | Mycobacterium avium subsp. paratuberculosis str. k10 | 4829781 | 4510 |
| NC_008146 | Mycobacterium sp. MCS | 5705448 | 5468 |
| NZ_CP010827 | Corynebacterium singulare strain IBS B52218 | 2830519 | 2556 |
| NZ_CP004350 | Corynebacterium casei LMG S-19264 | 3113488 | 2786 |
| NZ_CP009211 | Corynebacterium imitans strain DSM 44264 | 2565321 | 2336 |
| NZ_CP015186 | Corynebacterium pseudotuberculosis strain 36 | 2403412 | 2159 |

| | | | |
|---|---|---|---|
| NZ_CP012695 | Corynebacterium pseudotuberculosis strain PO269-5 | 2337124 | 2081 |
| NZ_CP009449 | Mycobacterium bovis strain ATCC BAA-935 | 4358088 | 4189 |
| NC_007164 | Corynebacterium jeikeium K411 complete genome. | 2462499 | 2116 |
| NZ_CP015495 | Mycobacterium avium subsp. paratuberculosis strain MAP/TANUVAS/TN/India/200 | 4829781 | 4501 |
| NZ_CP009251 | Corynebacterium stationis strain 622=DSM 20302 | 2808767 | 2520 |
| NC_012207 | Mycobacterium bovis BCG str. Tokyo 172 DNA | 4371711 | 4183 |
| NZ_CP019420 | Mycobacterium sp. MS1601 | 6407860 | 6073 |
| NZ_CP007722 | Corynebacterium glutamicum strain ATCC 21831 | 3176076 | 2933 |
| NC_016790 | Corynebacterium diphtheriae VA01 | 2395441 | 2211 |
| NZ_CP016335 | Corynebacterium glutamicum strain ATCC 13869 | 3296500 | 3024 |
| NC_002677 | Mycobacterium leprae TN chromosome | 3268203 | 1605 |
| NZ_CP015187 | Corynebacterium pseudotuberculosis strain 38 | 2403515 | 2161 |
| NZ_CP014998 | Corynebacterium pseudotuberculosis strain Cp13 | 2342237 | 2085 |
| NZ_CP016888 | Mycobacterium tuberculosis strain SCAID 252.0 | 4439387 | 4257 |
| NZ_CP002882 | Mycobacterium tuberculosis BT2 | 4401899 | 4228 |
| NZ_CP009613 | Mycobacterium abscessus subsp. bolletii strain MC1518 | 5049258 | 5003 |
| NZ_CP013327 | Corynebacterium pseudotuberculosis strain PA01 | 2337920 | 2077 |
| NZ_CP012150 | Mycobacterium goodii strain X7B | 7105933 | 6662 |
| NC_018101 | Corynebacterium ulcerans 0102 DNA | 2579188 | 2302 |
| NZ_CP017291 | Corynebacterium pseudotuberculosis strain MEX30 | 2368140 | 2122 |
| NZ_CP010330 | Mycobacterium tuberculosis strain F28 | 4421903 | 4253 |
| NC_016787 | Corynebacterium diphtheriae HC03 | 2478364 | 2284 |
| NC_004369 | Corynebacterium efficiens YS-314 DNA | 3147090 | 2819 |
| NC_020133 | Mycobacterium liflandii 128FXT | 6208955 | 5477 |
| NC_013441 | Gordonia bronchialis DSM 43247 | 5208602 | 4800 |
| NC_008595 | Mycobacterium avium 104 | 5475491 | 5147 |
| NC_008268 | Rhodococcus jostii RHA1 | 7804765 | 7197 |
| NZ_CP014941 | Rhodococcus sp. BH4 | 6314891 | 5787 |
| NZ_CP009101 | Mycobacterium tuberculosis strain ZMC13-88 | 4411515 | 4227 |
| NZ_CP012837 | Corynebacterium pseudotuberculosis strain 1002B | 2335107 | 2078 |
| NZ_CP018302 | Mycobacterium tuberculosis strain I0004000-1 | 4365724 | 4201 |
| NZ_CP007790 | Corynebacterium marinum DSM 44953 | 2607268 | 2406 |
| NZ_CP011541 | Corynebacterium epidermidicanis strain DSM 45586 | 2692072 | 2417 |
| NZ_CP009220 | Corynebacterium deserti GIMN1.010 | 2972149 | 2682 |
| NZ_CP015964 | Mycobacterium yongonense strain Asan 36912 | 5445538 | 5074 |
| NC_016768 | Mycobacterium tuberculosis KZN 4207 | 4394985 | 4220 |
| NZ_CP013698 | Corynebacterium pseudotuberculosis strain PO222/4-1 | 2337508 | 2081 |
| NZ_CP013697 | Corynebacterium pseudotuberculosis strain MEX25 | 2337529 | 2079 |
| NZ_CP019882 | Mycobacterium litorale strain F4 | 6103712 | 5762 |
| NC_009077 | Mycobacterium sp. JLS | 6048425 | 5794 |
| NZ_CP017292 | Corynebacterium pseudotuberculosis strain MEX31 | 2367880 | 2119 |
| NC_008705 | Mycobacterium sp. KMS | 5737227 | 5512 |
| NC_018612 | Mycobacterium indicus pranii MTCC 9506 | 5589007 | 5214 |
| NC_019950 | Mycobacterium canettii CIPT 140060008 complete genome. | 4432426 | 4219 |
| NZ_CP013263 | Corynebacterium pseudotuberculosis strain MB66 | 2372202 | 2127 |
| NZ_CP018363 | Mycobacterium avium subsp. hominissuis strain H87 | 5626623 | 5223 |
| NZ_CP009246 | Corynebacterium flavescens strain OJ8 | 2758653 | 2467 |
| NC_015673 | Corynebacterium resistens DSM 45100 | 2601311 | 2206 |
| NZ_CP012390 | Corynebacteriales bacterium X1698 | 1915154 | 1615 |
| NZ_AP014547 | Mycobacterium abscessus subsp. bolletii CCUG 48898 = JCM 15300 DNA | 4978382 | 4944 |
| NZ_CP017920 | Mycobacterium tuberculosis strain TB282 | 4425860 | 4257 |
| NC_016887 | Nocardia cyriacigeorgica GUH-2 chromosome complete genome. | 6194645 | 5544 |
| NC_008596 | Mycobacterium smegmatis str. MC2 155 chromosome | 6988209 | 6717 |
| NC_018143 | Mycobacterium tuberculosis H37Rv | 4411709 | 4235 |
| NZ_CP016594 | Gordonia terrae strain 3612 | 5701501 | 5035 |
| NZ_CP011312 | Corynebacterium kutscheri strain DSM 20755 | 2354065 | 2056 |
| NZ_CP011295 | Rhodococcus erythropolis strain BG43 | 6334075 | 5804 |
| NZ_CP018305 | Mycobacterium tuberculosis strain M0018684-2 | 4359825 | 4188 |
| NC_019952 | Mycobacterium canettii CIPT 140070017 complete genome. | 4524466 | 4269 |
| NC_009565 | Mycobacterium tuberculosis F11 | 4424435 | 4237 |
| NZ_CP014566 | Mycobacterium bovis BCG str. Tokyo 172 substrain TRCS | 4371707 | 4183 |
| NZ_CP007156 | Corynebacterium falsenii DSM 44353 strain BL 8171 | 2677607 | 2259 |
| NZ_CP015622 | Corynebacterium sp. JZ16 | 3047373 | 2733 |
| NC_018150 | Mycobacterium abscessus subsp. massiliense str. GO 06 | 4687873 | 4622 |
| NC_017301 | Corynebacterium pseudotuberculosis C231 | 2328208 | 2070 |
| NZ_CP014961 | Mycobacterium abscessus strain FLAC054 | 5330954 | 5355 |
| NZ_CP011545 | Corynebacterium testudinoris strain DSM 44614 | 2721226 | 2541 |
| NZ_CP008922 | Corynebacterium pseudotuberculosis strain 48252 | 2338139 | 2081 |
| NZ_CP009716 | Corynebacterium ulcerans strain 05146 | 2466435 | 2170 |
| NC_017031 | Corynebacterium pseudotuberculosis P54B96 | 2337657 | 2079 |
| NZ_CP008924 | Corynebacterium pseudotuberculosis strain Ft_2193/67 | 2338300 | 2081 |
| NZ_CP008944 | Corynebacterium atypicum strain R2070 | 2311380 | 2009 |
| NC_015683 | Corynebacterium ulcerans BR-AD22 | 2606374 | 2340 |
| NZ_CP019221 | Mycobacterium chimaera strain CDC 2015-22-71 | 6078402 | 5625 |
| NZ_CP014341 | Corynebacterium pseudotuberculosis strain E55 | 2335383 | 2076 |

| | | | |
|---|---|---|---|
| NC_017945 | Corynebacterium pseudotuberculosis 258 | 2369817 | 2126 |
| NZ_CP009496 | Mycobacterium smegmatis strain INHR2 | 6988302 | 6774 |
| NC_017522 | Mycobacterium tuberculosis CCDC5180 | 4405981 | 4233 |
| NC_017904 | Mycobacterium sp. MOTT36Y | 5613626 | 5234 |
| NC_012590 | Corynebacterium aurimucosum ATCC 700975 | 2790189 | 2563 |
| NC_016782 | Corynebacterium diphtheriae 241 | 2426551 | 2257 |
| NZ_CP015189 | Corynebacterium pseudotuberculosis strain 43 | 2365075 | 2116 |
| NC_018078 | Mycobacterium tuberculosis KZN 605 | 4399120 | 4225 |
| NZ_CP021252 | Corynebacterium striatum strain KC-Na-01 | 2758551 | 2550 |
| NZ_CP012194 | Corynebacterium glutamicum strain CP | 3342897 | 3072 |
| NC_022115 | Rhodococcus erythropolis CCM2595 | 6281198 | 5725 |
| NZ_CP012044 | Mycobacterium abscessus UC22 | 5257136 | 5226 |
| NZ_CP010797 | Rhodococcus sp. B7740 | 5341557 | 4956 |
| NZ_CP019705 | Corynebacterium ammoniagenes strain KCCM 40472 | 2808265 | 2498 |
| NZ_CP015192 | Corynebacterium pseudotuberculosis strain 34 | 2403454 | 2162 |
| NZ_CP013475 | Mycobacterium tuberculosis strain 1458 | 4402033 | 4226 |
| NC_012943 | Mycobacterium tuberculosis KZN 1435 | 4398250 | 4226 |
| NC_018289 | Mycobacterium smegmatis str. MC2 155 | 6988208 | 6787 |
| NZ_CP016826 | Corynebacterium pseudotuberculosis strain MEX29 | 2337866 | 2081 |
| NC_017305 | Corynebacterium pseudotuberculosis PAT10 | 2335323 | 2077 |
| NZ_CP014634 | Corynebacterium simulans strain PES1 | 2737971 | 2508 |
| NZ_CP011510 | Mycobacterium tuberculosis strain Beijing | 4378588 | 4199 |
| NZ_CP013049 | Mycobacterium abscessus strain NOV0213 | 5173145 | 5156 |
| NC_017524 | Mycobacterium tuberculosis CTRI-2 | 4398525 | 4226 |
| NZ_CP012022 | Corynebacterium pseudotuberculosis strain 262 | 2361125 | 2099 |
| NZ_CP015220 | Rhodococcus sp. PBTS2 | 5179353 | 4784 |
| NZ_CP014984 | Corynebacterium glutamicum strain YI | 3342103 | 3148 |
| NZ_CP004062 | Corynebacterium glutamicum ZL-6 | 3332458 | 3079 |
| NZ_CP016972 | Mycobacterium tuberculosis H37Ra | 4426109 | 4248 |
| NZ_CP018175 | Corynebacterium glutamicum strain XV | 3333639 | 3069 |
| NZ_CP014952 | Mycobacterium abscessus strain FLAC005 | 4869298 | 4772 |
| NZ_CP017839 | Nocardia seriolae strain EM150506 | 8304518 | 7637 |
| NZ_CP013261 | Corynebacterium pseudotuberculosis strain MB14 | 2370761 | 2127 |
| NZ_CP016191 | Mycobacterium abscessus strain FLAC030 | 4867257 | 4826 |
| NC_011896 | Mycobacterium leprae Br4923 | 3268071 | 2900 |
| NZ_CP009483 | Mycobacterium kansasii 824 | 6402301 | 5588 |
| NZ_CP015965 | Mycobacterium yongonense strain Asan 36527 | 5435152 | 5062 |
| NC_021351 | Corynebacterium glutamicum SCgG1 | 3350620 | 3050 |
| NZ_CP010071 | Mycobacterium sp. QIA-37 | 4855372 | 4673 |
| NZ_CP007724 | Corynebacterium glutamicum strain AR1 | 3145677 | 2912 |
| NZ_CP011269 | Mycobacterium fortuitum strain CT6 | 6254616 | 5950 |
| NZ_CP020821 | Mycobacterium colombiense CECT 3035 | 5581643 | 5236 |
| NC_016804 | Mycobacterium bovis BCG str. Mexico | 4350386 | 4162 |
| NZ_CP016396 | Mycobacterium avium strain RCAD0278 | 4953610 | 4595 |
| NZ_CP015191 | Corynebacterium pseudotuberculosis strain 48 | 2403301 | 2160 |
| NZ_CP009427 | Mycobacterium tuberculosis strain 96121 | 4410945 | 4232 |
| NZ_CP011022 | Mycobacterium sp. NRRL B-3805 | 5421338 | 5049 |
| NZ_CP012749 | Rhodococcus sp. 008 | 6570200 | 6024 |
| NC_009338 | Mycobacterium gilvum PYR-GCK | 5619607 | 5311 |
| NZ_CP017299 | Rhodococcus sp. YL-1 | 6367154 | 5845 |
| NC_012704 | Corynebacterium kroppenstedtii DSM 44385 | 2446804 | 2030 |
| NZ_CP017384 | Corynebacterium pseudotuberculosis strain I37 | 2370282 | 2108 |
| NZ_HG813240 | Mycobacterium tuberculosis 49-02 complete genome. | 4412379 | 4234 |
| NC_015564 | Amycolicicoccus subflavus DQS3-9A1 | 4738809 | 4371 |
| NZ_CP006841 | Corynebacterium lactis RW2-5 | 2769745 | 2405 |
| NZ_CP011341 | Rhodococcus aetherivorans strain IcdP1 | 5922748 | 5319 |
| NZ_CP012136 | Corynebacterium pseudotuberculosis strain E19 | 2367956 | 2120 |
| NC_020089 | Mycobacterium tuberculosis 7199-99 complete genome. | 4421197 | 4237 |
| NZ_CP009247 | Corynebacterium frankenforstense DSM 45800 strain ST18 | 2604152 | 2193 |
| NZ_CP014959 | Mycobacterium abscessus strain FLAC048 | 4939234 | 4879 |
| NZ_CP009215 | Corynebacterium ureicelerivorans strain IMMIB RIV-2301 | 2279990 | 2174 |
| NZ_CP018063 | Rhodococcus sp. 2G | 5231430 | 4866 |
| NZ_CP009914 | Mycobacterium sp. VKM Ac-1817D | 6324222 | 6024 |
| NC_002755 | Mycobacterium tuberculosis CDC1551 | 4403837 | 4231 |
| NC_022040 | Corynebacterium glutamicum MB001 | 3079253 | 2825 |
| NC_016785 | Corynebacterium diphtheriae CDCE 8392 | 2433326 | 2270 |
| NZ_CP019768 | Corynebacterium pseudotuberculosis strain phoP | 2339296 | 2088 |
| NC_014814 | Mycobacterium gilvum Spyr1 | 5547747 | 5235 |
| NZ_CP009494 | Mycobacterium smegmatis str. MC2 155 | 6988269 | 6774 |
| NC_023036 | Mycobacterium neoaurum VKM Ac-1815D | 5421267 | 5048 |
| NZ_CP009616 | Mycobacterium abscessus strain 4529 | 4687494 | 4623 |
| NZ_CP013699 | Corynebacterium pseudotuberculosis strain E56 | 2335773 | 2077 |
| NZ_CP015235 | Rhodococcus fascians D188 | 5139988 | 4747 |
| NZ_CP012506 | Mycobacterium tuberculosis strain SCAID 187.0 | 4411829 | 4234 |
| NZ_CP012885 | Mycobacterium chimaera strain AH16 | 5852822 | 5410 |

| | | | |
|---|---|---|---|
| NZ_CP010114 | Mycobacterium avium subsp. paratuberculosis strain E93 | 4786065 | 4507 |
| NZ_CP011546 | Corynebacterium uterequi strain DSM 45634 | 2419437 | 2150 |
| NZ_CP009407 | Mycobacterium abscessus subsp. bolletii 103 | 5051394 | 4995 |
| NZ_CP017594 | Mycobacterium tuberculosis strain Beijing-like/36918 | 4441591 | 4260 |
| NZ_CP016190 | Mycobacterium abscessus strain FLAC028 | 5188101 | 5151 |
| NC_022350 | Mycobacterium tuberculosis str. Haarlem | 4408224 | 4220 |
| NZ_AP012555 | Mycobacterium avium subsp. hominissuis TH135 chromosomal DNA | 4951217 | 4581 |
| NZ_CP013991 | Corynebacterium glutamicum strain USDA-ARS-USMARC-56828 | 3245395 | 2976 |
| NC_019951 | Mycobacterium canettii CIPT 140070010 complete genome. | 4525948 | 4270 |
| NZ_CP007803 | Mycobacterium tuberculosis K | 4385518 | 4214 |
| NZ_CP004046 | [Brevibacterium] flavum ZL-1 | 3340941 | 3090 |
| NC_017300 | Corynebacterium pseudotuberculosis 1002 | 2335113 | 2078 |
| NC_015758 | Mycobacterium africanum GM041182 complete genome. | 4389314 | 4192 |
| NZ_CP016819 | Rhodococcus sp. p52 | 4893347 | 4535 |
| NC_017308 | Corynebacterium pseudotuberculosis 1/06-A | 2279118 | 2037 |
| NC_016802 | Corynebacterium diphtheriae HC02 | 2468612 | 2310 |
| NZ_CP019587 | Corynebacterium pseudotuberculosis strain PA04 | 2338093 | 2078 |
| NZ_CP014950 | Mycobacterium abscessus strain FLAC003 | 4826045 | 4714 |
| NC_016783 | Corynebacterium diphtheriae INCA 402 | 2449071 | 2279 |
| NC_003450 | Corynebacterium glutamicum ATCC 13032 chromosome | 3309401 | 2959 |
| NZ_CP014958 | Mycobacterium abscessus strain FLAC045 | 5217908 | 5256 |
| NZ_CP009495 | Mycobacterium smegmatis strain INHR1 | 6988337 | 6774 |
| NZ_CP021251 | Corynebacterium pseudotuberculosis strain ATCC 19410 | 2337763 | 2084 |
| NC_016947 | Mycobacterium intracellulare MOTT-02 | 5409696 | 5059 |
| NZ_CP010451 | Corynebacterium glutamicum strain B253 | 3207539 | 2932 |
| NZ_CP012095 | Mycobacterium bovis strain 1595 | 4351712 | 4169 |
| NZ_CP006842 | Corynebacterium glyciniphilum AJ 3170 | 3509786 | 3201 |
| NC_016934 | Mycobacterium tuberculosis UT205 complete genome. | 4418088 | 4195 |
| NC_014329 | Corynebacterium pseudotuberculosis FRC41 | 2337913 | 2080 |
| NZ_CP009408 | Mycobacterium abscessus subsp. bolletii strain MA 1948 | 5064190 | 5018 |
| NZ_CP007220 | Mycobacterium chelonae CCUG 47445 | 5029817 | 4867 |
| NZ_CP009493 | Mycobacterium avium subsp. avium 2285 (R) | 5169415 | 4851 |
| NZ_CP009482 | Mycobacterium avium subsp. avium 2285 (S) | 5197664 | 4856 |
| NZ_CP017593 | Mycobacterium tuberculosis strain Beijing-like/35049 | 4427062 | 4250 |
| NZ_AP014573 | Mycobacterium tuberculosis str. Kurono DNA | 4415078 | 4240 |
| NZ_CP006764 | Corynebacterium doosanense CAU 212 = DSM 45436 | 2671798 | 2542 |
| NC_006958 | Corynebacterium glutamicum ATCC 13032 | 3282708 | 3017 |
| NZ_CP018304 | Mycobacterium tuberculosis strain M0002959-6 | 4386447 | 4208 |
| NZ_CP011491 | Mycobacterium vaccae 95051 | 6235754 | 5826 |
| NC_021251 | Mycobacterium tuberculosis CCDC5079 | 4414325 | 4234 |
| NC_016948 | Mycobacterium intracellulare MOTT-64 | 5501090 | 5158 |
| NZ_CP009248 | Corynebacterium sphenisci DSM 44792 | 2594799 | 2283 |
| NZ_CP017595 | Mycobacterium tuberculosis strain Beijing-like/38774 | 4431885 | 4259 |
| NC_009342 | Corynebacterium glutamicum R DNA | 3314179 | 3046 |
| NZ_CP018300 | Mycobacterium tuberculosis strain I0002353-6 | 4385578 | 4208 |
| NC_016786 | Corynebacterium diphtheriae HC01 | 2427149 | 2257 |
| NZ_CP009426 | Mycobacterium tuberculosis strain 96075 | 4379376 | 4218 |
| NC_016800 | Corynebacterium diphtheriae BH8 | 2485519 | 2399 |
| NC_021715 | Mycobacterium sp. 05-1390 | 5521023 | 5142 |
| NZ_CP009622 | Corynebacterium ulcerans FRC11 | 2442826 | 2143 |
| NZ_CP008923 | Corynebacterium pseudotuberculosis strain CS_10 | 2338144 | 2082 |
| NZ_CP020381 | Mycobacterium tuberculosis strain MTB1 | 4433542 | 4255 |
| NZ_CP010795 | Corynebacterium pseudotuberculosis strain 29156 | 2338645 | 2082 |
| NC_020559 | Mycobacterium tuberculosis str. Erdman = ATCC 35801 DNA | 4392353 | 4233 |
| NZ_CP011311 | Corynebacterium camporealensis strain DSM 44610 | 2451810 | 2223 |
| NC_021194 | Mycobacterium tuberculosis EAI5/NITR206 | 4390306 | 4221 |
| NZ_CP015219 | Rhodococcus sp. PBTS1 | 4251687 | 3861 |
| NC_017306 | Corynebacterium pseudotuberculosis 42/02-A | 2337606 | 2079 |
| NZ_CP014475 | Mycobacterium phlei strain CCUG 21000 | 5349645 | 5090 |
| NC_021282 | Mycobacterium abscessus subsp. bolletii 50594 | 5000473 | 5011 |
| NC_016781 | Corynebacterium pseudotuberculosis 3/99-5 | 2337938 | 2080 |
| NC_016801 | Corynebacterium diphtheriae C7 (beta) | 2499189 | 2372 |
| NC_010612 | Mycobacterium marinum M | 6636827 | 5588 |
| NZ_CP013260 | Corynebacterium pseudotuberculosis strain MB11 | 2363423 | 2124 |
| NZ_CP015188 | Corynebacterium pseudotuberculosis strain 39 | 2403579 | 2159 |
| NZ_CP015100 | Corynebacterium pseudotuberculosis strain T1 | 2337201 | 2079 |
| NZ_CP015596 | Mycobacterium sp. YC-RL4 | 5801417 | 5519 |
| NZ_CP009614 | Mycobacterium avium subsp. avium strain DJO-44271 | 5011264 | 4683 |
| NZ_CP019769 | Corynebacterium pseudotuberculosis strain MIC6 | 2337147 | 2079 |
| NZ_CP014954 | Mycobacterium abscessus strain FLAC008 | 5166100 | 5177 |
| NC_015848 | Mycobacterium canettii CIPT 140010059 complete genome. | 4482059 | 4242 |
| NZ_CP011474 | Corynebacterium pseudotuberculosis strain 12C | 2337451 | 2080 |
| NZ_CP018043 | Mycobacterium sp. WY10 | 6041408 | 5846 |
| NC_016932 | Corynebacterium pseudotuberculosis 316 | 2310415 | 2057 |
| NC_015859 | Corynebacterium variabile DSM 44702 | 3433007 | 3087 |

| | | | |
|---|---|---|---|
| NZ_CP006850 | Nocardia nova SH22a | 8348532 | 7504 |
| NZ_CP017596 | Mycobacterium tuberculosis strain Beijing/391 | 4406925 | 4246 |
| NZ_CP015185 | Corynebacterium pseudotuberculosis strain 35 | 2403502 | 2162 |
| NZ_CP021122 | Mycobacterium abscessus subsp. massiliense strain FLAC047 | 4936470 | 4851 |
| NZ_CP014543 | Corynebacterium pseudotuberculosis strain MEX9 | 2337578 | 2082 |
| NZ_CP002871 | Mycobacterium tuberculosis HKBS1 | 4407929 | 4235 |
| NC_021740 | Mycobacterium tuberculosis EAI5 | 4391174 | 4210 |
| NZ_CP010339 | Mycobacterium tuberculosis strain 22103 | 4399422 | 4222 |
| NZ_CP015183 | Corynebacterium pseudotuberculosis strain 32 | 2403533 | 2159 |
| NC_017307 | Corynebacterium pseudotuberculosis CIP 52.97 | 2369387 | 2124 |
| NC_019966 | Mycobacterium sp. JS623 | 6464916 | 6302 |
| NZ_CP012090 | Mycobacterium tuberculosis W-148 | 4418548 | 4240 |
| NZ_CP007809 | Mycobacterium tuberculosis strain KIT87190 | 4410788 | 4234 |
| NC_020230 | Corynebacterium urealyticum DSM 7111 | 2316065 | 1954 |
| NZ_CP017711 | Corynebacterium pseudotuberculosis strain MEX1 | 2337090 | 2081 |
| NC_016789 | Corynebacterium diphtheriae PW8 | 2530683 | 2383 |
| NC_020506 | Corynebacterium callunae DSM 20147 | 2839551 | 2558 |
| NC_012490 | Rhodococcus erythropolis PR4 DNA | 6516310 | 6020 |
| NZ_CP009499 | Mycobacterium intracellulare 1956 | 5183048 | 4855 |
| NZ_CP002885 | Mycobacterium tuberculosis CCDC5180 | 4414346 | 4238 |
| NZ_CP014960 | Mycobacterium abscessus strain FLAC049 | 4799801 | 4704 |
| NC_022663 | Mycobacterium kansasii ATCC 12478 | 6432277 | 5579 |
| NZ_CP018082 | Nocardia soli strain Y48 | 7310115 | 6524 |
| NZ_CP020356 | Corynebacterium pseudotuberculosis strain SigmaE | 2339255 | 2083 |
| NC_016788 | Corynebacterium diphtheriae HC04 | 2484332 | 2300 |
| NZ_CP009500 | Corynebacterium ulcerans strain 210932 | 2484335 | 2190 |
| NZ_CP011913 | Corynebacterium ulcerans FRC58 | 2542597 | 2267 |
| NZ_CP018331 | Corynebacterium diphtheriae strain B-D-16-78 | 2474151 | 2308 |
| NC_020519 | Corynebacterium glutamicum K051 complete genome | 3309400 | 3051 |
| NZ_CP011853 | Gordonia sp. QH-11 | 4428727 | 4093 |
| NZ_CP010337 | Mycobacterium tuberculosis strain 22115 | 4401829 | 4243 |
| NZ_CP011773 | Mycobacterium sp. EPa45 | 6177406 | 5834 |
| NZ_CP016189 | Mycobacterium immunogenum strain FLAC016 | 5604845 | 5488 |
| NZ_CP015184 | Corynebacterium pseudotuberculosis strain 33 | 2403550 | 2160 |
| NZ_CP011542 | Corynebacterium mustelae strain DSM 45274 | 3391554 | 2982 |
| NZ_CP009249 | Corynebacterium phocae strain M408/89/1 | 2779609 | 2441 |
| NZ_CP017597 | Mycobacterium tuberculosis strain Beijing-like/50148 | 4444417 | 4266 |
| NC_012522 | Rhodococcus opacus B4 DNA | 7913450 | 7222 |
| NC_022198 | Corynebacterium argentoratense DSM 44202 | 2031902 | 1842 |
| NC_006361 | Nocardia farcinica IFM 10152 DNA | 6021225 | 5600 |
| NZ_CP008913 | Corynebacterium sp. ATCC 6931 | 2471920 | 2088 |
| NZ_CP013146 | Corynebacterium pseudotuberculosis strain N1 | 2337845 | 2078 |
| NZ_CP011530 | Mycobacterium immunogenum strain CCUG 47286 | 5573781 | 5466 |
| NZ_CP009111 | Rhodococcus opacus strain 1CP | 7687653 | 7037 |
| NZ_CP021417 | Corynebacterium ulcerans strain PO100/5 | 2572413 | 2332 |
| NZ_CP018778 | Mycobacterium tuberculosis strain DK9897 | 4411511 | 4232 |
| NZ_CP016193 | Mycobacterium abscessus strain FLAC055 | 5331134 | 5356 |
| NC_015576 | Mycobacterium sinense strain JDM601 | 4643668 | 4358 |
| NC_021200 | Mycobacterium avium subsp. paratuberculosis MAP4 | 4829424 | 4500 |
| NZ_CP011309 | [Brevibacterium] flavum strain ATCC 15168 | 3338699 | 3141 |
| NZ_CP011095 | Corynebacterium ulcerans strain 131002 | 2434569 | 2140 |
| NZ_CP017598 | Mycobacterium tuberculosis strain Beijing-like/1104 | 4380156 | 4220 |
| NZ_CP009927 | Corynebacterium pseudotuberculosis strain VD57 | 2337177 | 2077 |
| NZ_CP009447 | Mycobacterium abscessus subsp. bolletii strain MM1513 | 4501725 | 4427 |
| NZ_CP016794 | Mycobacterium tuberculosis strain SCAID 320.0 | 4406628 | 4250 |
| NC_018027 | Mycobacterium chubuense NBB4 | 5583723 | 5226 |
| NZ_CP015190 | Corynebacterium pseudotuberculosis strain 46 | 2366565 | 2115 |
| NZ_CP014953 | Mycobacterium abscessus strain FLAC007 | 5064478 | 5053 |
| NZ_CP003949 | Rhodococcus opacus PD630 | 8376953 | 7603 |
| NC_017317 | Corynebacterium ulcerans 809 | 2502095 | 2208 |
| NZ_CP019963 | Corynebacterium stationis strain LMG 21670 | 2871159 | 2601 |
| NZ_CP014957 | Mycobacterium abscessus strain FLAC031 | 5146255 | 5061 |
| NZ_CP009243 | Mycobacterium bovis BCG strain Russia 368 | 4370138 | 4182 |
| NC_016799 | Corynebacterium diphtheriae 31A | 2535346 | 2411 |
| NZ_CP002883 | Mycobacterium tuberculosis BT1 | 4399405 | 4227 |
| NZ_CP019572 | Rhodococcus sp. MTM3W5.2 | 5665081 | 5101 |
| NC_014158 | Tsukamurella paurometabola DSM 20162 | 4379918 | 4177 |
| NZ_CP009100 | Mycobacterium tuberculosis strain ZMC13-264 | 4411507 | 4225 |
| NZ_CP015309 | Corynebacterium pseudotuberculosis strain PA02 | 2328435 | 2070 |

Additional file A3 - Number of Genomic Islands (GI) and Putative Transferred Genes (PTG) per genome

| Genome ID | Number of GI | Number of PTG |
|---|---|---|
| NC_002677 | 0 | 0 |
| NC_014329 | 0 | 0 |
| NC_016781 | 0 | 0 |
| NC_016932 | 0 | 0 |
| NC_017031 | 0 | 0 |
| NC_017300 | 0 | 0 |
| NC_017301 | 0 | 0 |
| NC_017303 | 0 | 0 |
| NC_017305 | 0 | 0 |
| NC_017306 | 0 | 0 |
| NC_017308 | 0 | 0 |
| NC_017462 | 0 | 0 |
| NZ_CP008922 | 0 | 0 |
| NZ_CP008923 | 0 | 0 |
| NZ_CP008924 | 0 | 0 |
| NZ_CP009927 | 0 | 0 |
| NZ_CP010795 | 0 | 0 |
| NZ_CP010889 | 0 | 0 |
| NZ_CP011474 | 0 | 0 |
| NZ_CP012695 | 0 | 0 |
| NZ_CP012837 | 0 | 0 |
| NZ_CP013146 | 0 | 0 |
| NZ_CP013327 | 0 | 0 |
| NZ_CP013697 | 0 | 0 |
| NZ_CP013698 | 0 | 0 |
| NZ_CP013699 | 0 | 0 |
| NZ_CP014341 | 0 | 0 |
| NZ_CP014543 | 0 | 0 |
| NZ_CP014998 | 0 | 0 |
| NZ_CP015100 | 0 | 0 |
| NZ_CP015309 | 0 | 0 |
| NZ_CP016826 | 0 | 0 |
| NZ_CP017711 | 0 | 0 |
| NZ_CP019768 | 0 | 0 |
| NZ_CP019769 | 0 | 0 |
| NZ_CP020356 | 0 | 0 |
| NZ_CP021251 | 0 | 0 |
| NZ_CP011312 | 1 | 172 |
| NZ_CP012022 | 1 | 942 |
| NZ_CP012136 | 1 | 9 |
| NZ_CP014960 | 1 | 23 |
| NZ_CP017384 | 1 | 11 |
| NZ_CP019587 | 1 | 13 |

| | | |
|---|---|---|
| NC_011896 | 2 | 38 |
| NC_022115 | 2 | 37 |
| NZ_CP009312 | 2 | 72 |
| NZ_CP009447 | 2 | 35 |
| NZ_CP009500 | 2 | 65 |
| NZ_CP009622 | 2 | 40 |
| NZ_CP009716 | 2 | 41 |
| NZ_CP010071 | 2 | 2033 |
| NZ_CP010818 | 2 | 65 |
| NZ_CP011095 | 2 | 42 |
| NZ_CP011913 | 2 | 107 |
| NZ_CP012390 | 2 | 120 |
| NZ_CP016829 | 2 | 18 |
| NZ_CP017291 | 2 | 21 |
| NC_006958 | 3 | 205 |
| NC_012704 | 3 | 80 |
| NC_015683 | 3 | 174 |
| NC_017307 | 3 | 32 |
| NC_017317 | 3 | 265 |
| NC_017945 | 3 | 30 |
| NC_018101 | 3 | 427 |
| NC_020519 | 3 | 198 |
| NC_022040 | 3 | 31 |
| NC_022198 | 3 | 1039 |
| NZ_CP008913 | 3 | 227 |
| NZ_CP008944 | 3 | 143 |
| NZ_CP009499 | 3 | 44 |
| NZ_CP009583 | 3 | 247 |
| NZ_CP011542 | 3 | 81 |
| NZ_CP013260 | 3 | 31 |
| NZ_CP013261 | 3 | 30 |
| NZ_CP013262 | 3 | 107 |
| NZ_CP013263 | 3 | 29 |
| NZ_CP015183 | 3 | 348 |
| NZ_CP015184 | 3 | 358 |
| NZ_CP015186 | 3 | 355 |
| NZ_CP015187 | 3 | 358 |
| NZ_CP015188 | 3 | 354 |
| NZ_CP015189 | 3 | 30 |
| NZ_CP015190 | 3 | 29 |
| NZ_CP015191 | 3 | 470 |
| NZ_CP015192 | 3 | 355 |
| NZ_CP015622 | 3 | 94 |
| NZ_CP017292 | 3 | 33 |
| NZ_CP019705 | 3 | 634 |
| NC_009342 | 4 | 651 |
| NC_015576 | 4 | 137 |
| NC_016790 | 4 | 336 |
| NC_017730 | 4 | 363 |
| NZ_CP007255 | 4 | 155 |
| NZ_CP009211 | 4 | 417 |
| NZ_CP009244 | 4 | 69 |

| | | |
|---|---|---|
| NZ_CP009248 | 4 | 82 |
| NZ_CP009251 | 4 | 1221 |
| NZ_CP009407 | 4 | 580 |
| NZ_CP009408 | 4 | 725 |
| NZ_CP009613 | 4 | 680 |
| NZ_CP011341 | 4 | 91 |
| NZ_CP011545 | 4 | 122 |
| NZ_CP014634 | 4 | 127 |
| NZ_CP014635 | 4 | 61 |
| NZ_CP014952 | 4 | 71 |
| NZ_CP014955 | 4 | 616 |
| NZ_CP015185 | 4 | 371 |
| NZ_CP015235 | 4 | 46 |
| NZ_CP016188 | 4 | 274 |
| NZ_CP018331 | 4 | 127 |
| NZ_CP021417 | 4 | 447 |
| NC_012590 | 5 | 435 |
| NC_016782 | 5 | 480 |
| NC_016786 | 5 | 471 |
| NC_016801 | 5 | 619 |
| NC_018150 | 5 | 117 |
| NC_018581 | 5 | 157 |
| NC_021351 | 5 | 449 |
| NC_021352 | 5 | 448 |
| NC_021915 | 5 | 320 |
| NZ_CP004350 | 5 | 654 |
| NZ_CP009215 | 5 | 228 |
| NZ_CP009220 | 5 | 123 |
| NZ_CP009245 | 5 | 313 |
| NZ_CP009247 | 5 | 63 |
| NZ_CP009615 | 5 | 118 |
| NZ_CP009616 | 5 | 117 |
| NZ_CP010113 | 5 | 68 |
| NZ_CP011311 | 5 | 257 |
| NZ_CP013991 | 5 | 240 |
| NZ_CP014941 | 5 | 3103 |
| NZ_CP014950 | 5 | 171 |
| NZ_CP014984 | 5 | 178 |
| NZ_CP020410 | 5 | 267 |
| NZ_CP020821 | 5 | 99 |
| NC_002935 | 6 | 581 |
| NC_014168 | 6 | 607 |
| NC_016788 | 6 | 346 |
| NC_016789 | 6 | 1190 |
| NC_018612 | 6 | 263 |
| NZ_CP006764 | 6 | 774 |
| NZ_CP006842 | 6 | 88 |
| NZ_CP007722 | 6 | 514 |
| NZ_CP007724 | 6 | 489 |
| NZ_CP009914 | 6 | 913 |
| NZ_CP010451 | 6 | 161 |
| NZ_CP011309 | 6 | 207 |
| NZ_CP011541 | 6 | 359 |

| | | |
|---|---|---|
| NZ_CP011883 | 6 | 3115 |
| NZ_CP012194 | 6 | 209 |
| NZ_CP013049 | 6 | 809 |
| NZ_CP014956 | 6 | 771 |
| NZ_CP014959 | 6 | 302 |
| NZ_CP015220 | 6 | 335 |
| NZ_CP015529 | 6 | 94 |
| NZ_CP015964 | 6 | 245 |
| NZ_CP016190 | 6 | 771 |
| NZ_CP016191 | 6 | 627 |
| NZ_CP016335 | 6 | 219 |
| NZ_CP020658 | 6 | 432 |
| NC_003450 | 7 | 287 |
| NC_016802 | 7 | 1030 |
| NC_016948 | 7 | 301 |
| NC_018027 | 7 | 205 |
| NC_020230 | 7 | 111 |
| NC_023036 | 7 | 1235 |
| NZ_CP005286 | 7 | 171 |
| NZ_CP006841 | 7 | 248 |
| NZ_CP007156 | 7 | 179 |
| NZ_CP007220 | 7 | 212 |
| NZ_CP007790 | 7 | 158 |
| NZ_CP009249 | 7 | 205 |
| NZ_CP011295 | 7 | 144 |
| NZ_CP011491 | 7 | 431 |
| NZ_CP011546 | 7 | 104 |
| NZ_CP011773 | 7 | 94 |
| NZ_CP014279 | 7 | 850 |
| NZ_CP014957 | 7 | 466 |
| NZ_CP014961 | 7 | 1367 |
| NZ_CP015961 | 7 | 928 |
| NZ_CP015965 | 7 | 259 |
| NZ_CP016193 | 7 | 1368 |
| NZ_CP016396 | 7 | 105 |
| NZ_CP017014 | 7 | 1671 |
| NZ_CP018175 | 7 | 227 |
| NZ_CP019963 | 7 | 593 |
| NC_016604 | 8 | 298 |
| NC_016783 | 8 | 380 |
| NC_016785 | 8 | 704 |
| NC_016787 | 8 | 628 |
| NC_016799 | 8 | 909 |
| NC_016946 | 8 | 154 |
| NC_019966 | 8 | 268 |
| NC_020302 | 8 | 678 |
| NC_020506 | 8 | 337 |
| NC_021663 | 8 | 396 |
| NC_023150 | 8 | 379 |
| NZ_AP014547 | 8 | 766 |
| NZ_CP010797 | 8 | 101 |
| NZ_CP011022 | 8 | 1244 |
| NZ_CP012044 | 8 | 559 |

| | | |
|---|---|---|
| NZ_CP012885 | 8 | 353 |
| NZ_CP016640 | 8 | 347 |
| NZ_CP016819 | 8 | 356 |
| NZ_CP017299 | 8 | 3111 |
| NZ_CP019572 | 8 | 811 |
| NZ_CP019882 | 8 | 266 |
| NZ_CP021122 | 8 | 718 |
| NC_000962 | 9 | 157 |
| NC_006361 | 9 | 3807 |
| NC_007164 | 9 | 401 |
| NC_012490 | 9 | 3313 |
| NC_012522 | 9 | 5191 |
| NC_016800 | 9 | 850 |
| NC_016947 | 9 | 116 |
| NZ_AP012555 | 9 | 305 |
| NZ_CP009482 | 9 | 544 |
| NZ_CP009614 | 9 | 487 |
| NZ_CP010114 | 9 | 203 |
| NZ_CP014953 | 9 | 1121 |
| NZ_CP015219 | 9 | 261 |
| NZ_CP016594 | 9 | 736 |
| NZ_CP018305 | 9 | 162 |
| NC_008769 | 10 | 179 |
| NC_009077 | 10 | 466 |
| NC_014158 | 10 | 1178 |
| NC_015564 | 10 | 473 |
| NC_018681 | 10 | 436 |
| NC_019965 | 10 | 210 |
| NC_020245 | 10 | 181 |
| NC_021282 | 10 | 1244 |
| NZ_CP004046 | 10 | 282 |
| NZ_CP004062 | 10 | 265 |
| NZ_CP004353 | 10 | 671 |
| NZ_CP009493 | 10 | 551 |
| NZ_CP010827 | 10 | 596 |
| NZ_CP012095 | 10 | 175 |
| NZ_CP014475 | 10 | 397 |
| NZ_CP014958 | 10 | 1298 |
| NZ_CP016189 | 10 | 1593 |
| NZ_CP018778 | 10 | 213 |
| NZ_CP019221 | 10 | 648 |
| NC_004369 | 11 | 738 |
| NC_012207 | 11 | 216 |
| NC_015673 | 11 | 1373 |
| NC_015848 | 11 | 370 |
| NC_016804 | 11 | 195 |
| NC_016887 | 11 | 354 |
| NZ_AM412059 | 11 | 214 |
| NZ_CP003494 | 11 | 442 |
| NZ_CP006850 | 11 | 251 |
| NZ_CP009243 | 11 | 216 |
| NZ_CP009449 | 11 | 212 |
| NZ_CP011530 | 11 | 1755 |

| | | |
|---|---|---|
| NZ_CP014566 | 11 | 216 |
| NZ_CP018300 | 11 | 219 |
| NZ_CP021252 | 11 | 574 |
| NC_002755 | 12 | 252 |
| NC_009338 | 12 | 662 |
| NC_015758 | 12 | 214 |
| NC_021200 | 12 | 227 |
| NC_021715 | 12 | 375 |
| NC_021740 | 12 | 228 |
| NC_022350 | 12 | 233 |
| NZ_CP008744 | 12 | 232 |
| NZ_CP009246 | 12 | 1027 |
| NZ_CP009427 | 12 | 1019 |
| NZ_CP009483 | 12 | 989 |
| NZ_CP011269 | 12 | 362 |
| NZ_CP011853 | 12 | 3630 |
| NZ_CP012506 | 12 | 263 |
| NZ_CP013741 | 12 | 231 |
| NZ_CP014951 | 12 | 1372 |
| NZ_CP014954 | 12 | 1199 |
| NZ_CP015773 | 12 | 219 |
| NZ_CP018302 | 12 | 248 |
| NZ_CP018303 | 12 | 231 |
| NZ_CP018304 | 12 | 232 |
| NZ_CP020381 | 12 | 247 |
| NC_002944 | 13 | 2262 |
| NC_008595 | 13 | 793 |
| NC_014814 | 13 | 1866 |
| NC_015859 | 13 | 248 |
| NC_016934 | 13 | 269 |
| NC_017904 | 13 | 420 |
| NC_018143 | 13 | 250 |
| NC_019950 | 13 | 385 |
| NC_020089 | 13 | 257 |
| NC_021194 | 13 | 267 |
| NC_022663 | 13 | 1001 |
| NZ_AP014573 | 13 | 266 |
| NZ_CP002882 | 13 | 285 |
| NZ_CP003949 | 13 | 496 |
| NZ_CP007027 | 13 | 249 |
| NZ_CP007803 | 13 | 263 |
| NZ_CP009100 | 13 | 228 |
| NZ_CP009101 | 13 | 253 |
| NZ_CP009426 | 13 | 276 |
| NZ_CP010337 | 13 | 296 |
| NZ_CP010339 | 13 | 267 |
| NZ_CP012150 | 13 | 372 |
| NZ_CP015495 | 13 | 2264 |
| NZ_CP016888 | 13 | 267 |
| NZ_CP016972 | 13 | 237 |
| NZ_CP017597 | 13 | 292 |
| NC_010612 | 14 | 272 |
| NC_013441 | 14 | 2842 |

| | | |
|---|---|---|
| NC_021251 | 14 | 290 |
| NZ_CP002871 | 14 | 296 |
| NZ_CP002883 | 14 | 298 |
| NZ_CP002885 | 14 | 293 |
| NZ_CP009480 | 14 | 291 |
| NZ_CP011510 | 14 | 599 |
| NZ_CP012749 | 14 | 387 |
| NZ_CP017594 | 14 | 289 |
| NZ_CP017596 | 14 | 331 |
| NZ_CP017598 | 14 | 292 |
| NZ_CP017920 | 14 | 624 |
| NZ_CP018301 | 14 | 283 |
| NZ_HG813240 | 14 | 291 |
| NC_009565 | 15 | 297 |
| NC_010545 | 15 | 456 |
| NC_016906 | 15 | 1176 |
| NC_019951 | 15 | 212 |
| NZ_CP007809 | 15 | 304 |
| NZ_CP012090 | 15 | 313 |
| NZ_CP015596 | 15 | 2072 |
| NZ_CP017595 | 15 | 300 |
| NZ_CP018363 | 15 | 409 |
| NZ_CP020809 | 15 | 394 |
| NC_008146 | 16 | 507 |
| NC_012943 | 16 | 311 |
| NC_018078 | 16 | 310 |
| NC_020559 | 16 | 832 |
| NC_021054 | 16 | 351 |
| NZ_CP009111 | 16 | 553 |
| NZ_CP010330 | 16 | 324 |
| NZ_CP016192 | 16 | 914 |
| NZ_CP016794 | 16 | 325 |
| NZ_CP019420 | 16 | 647 |
| NC_009525 | 17 | 287 |
| NC_016768 | 17 | 330 |
| NC_017522 | 17 | 362 |
| NC_017524 | 17 | 310 |
| NZ_CP013475 | 17 | 1364 |
| NZ_CP017593 | 17 | 354 |
| NC_008705 | 18 | 3046 |
| NC_019952 | 18 | 311 |
| NC_020133 | 19 | 682 |
| NZ_CP018043 | 19 | 887 |
| NC_008596 | 20 | 402 |
| NC_008726 | 20 | 1277 |
| NZ_CP018063 | 20 | 1017 |
| NZ_CP009494 | 21 | 448 |
| NZ_CP009495 | 21 | 448 |
| NZ_CP009496 | 21 | 448 |
| NC_018289 | 22 | 477 |
| NZ_CP018082 | 22 | 846 |
| NC_008268 | 26 | 2162 |
| NZ_CP017839 | 46 | 1366 |

| | | |
|---|---|---|
| Total | 2874 | 168724 |

Additional file A4 - Statistics on homology groups

| Description | Numbers |
|---|---|
| Number of genes | 1368128 |
| Number of genes in homology groups | 1356782 |
| Number of unassigned genes | 11346 |
| Percentage of genes in homology groups | 99,2 |
| Percentage of unassigned genes | 0,8 |
| Number of homology groups | 17821 |
| Number of species-specific homology groups | 142 |
| Number of genes in species-specific homology groups | 365 |
| Percentage of genes in species-specific homology groups | 0 |
| Mean homology group size | 76,1 |
| Median homology group size | 10 |
| G50 (assigned genes) | 352 |
| G50 (all genes) | 349 |
| O50 (assigned genes) | 1318 |
| O50 (all genes) | 1334 |
| Number of homology groups with all species present | 188 |
| Number of single-copy homology groups | 13 |

| Composition of homology groups | Number of homology groups |
|---|---|
| 1 | 142 |
| 2 | 3197 |
| 3 | 1596 |
| 4 | 1166 |
| 5 | 972 |
| 6 | 595 |
| 7 | 594 |
| 8 | 484 |
| 9 | 321 |
| 10 | 331 |
| 11 | 253 |
| 12 | 190 |
| 13 | 185 |
| 14 | 169 |
| 15 | 167 |
| 16 | 126 |
| 17 | 139 |
| 18 | 120 |
| 19 | 126 |
| 20 | 94 |
| 21 | 112 |
| 22 | 93 |
| 23 | 118 |
| 24 | 104 |
| 25 | 83 |
| 26 | 66 |
| 27 | 77 |
| 28 | 87 |
| 29 | 78 |
| 30 | 76 |
| 31 | 93 |
| 32 | 76 |
| 33 | 140 |
| 34 | 79 |
| 35 | 77 |
| 36 | 58 |
| 37 | 62 |
| 38 | 47 |
| 39 | 37 |
| 40 | 33 |
| 41 | 42 |
| 42 | 41 |
| 43 | 49 |
| 44 | 31 |
| 45 | 38 |
| 46 | 36 |
| 47 | 33 |
| 48 | 27 |
| 49 | 40 |
| 50 | 37 |
| 51 | 32 |
| 52 | 32 |
| 53 | 37 |
| 54 | 35 |
| 55 | 35 |
| 56 | 30 |
| 57 | 34 |
| 58 | 26 |
| 59 | 38 |
| 60 | 39 |
| 61 | 27 |
| 62 | 31 |
| 63 | 33 |
| 64 | 21 |
| 65 | 25 |
| 66 | 25 |
| 67 | 21 |
| 68 | 25 |
| 69 | 33 |
| 70 | 54 |
| 71 | 52 |
| 72 | 50 |
| 73 | 76 |
| 74 | 81 |
| 75 | 97 |
| 76 | 87 |
| 77 | 60 |
| 78 | 43 |
| 79 | 46 |
| 80 | 29 |
| 81 | 34 |
| 82 | 23 |
| 83 | 31 |
| 84 | 24 |
| 85 | 21 |
| 86 | 30 |
| 87 | 32 |
| 88 | 26 |
| 89 | 20 |
| 90 | 33 |
| 91 | 18 |
| 92 | 20 |
| 93 | 19 |
| 94 | 17 |
| 95 | 23 |
| 96 | 20 |
| 97 | 17 |
| 98 | 15 |
| 99 | 13 |
| 100 | 21 |
| 101 | 17 |
| 102 | 17 |
| 103 | 19 |
| 104 | 16 |
| 105 | 23 |
| 106 | 24 |
| 107 | 19 |
| 108 | 20 |
| 109 | 19 |
| 110 | 12 |
| 111 | 21 |
| 112 | 16 |
| 113 | 15 |
| 114 | 9 |

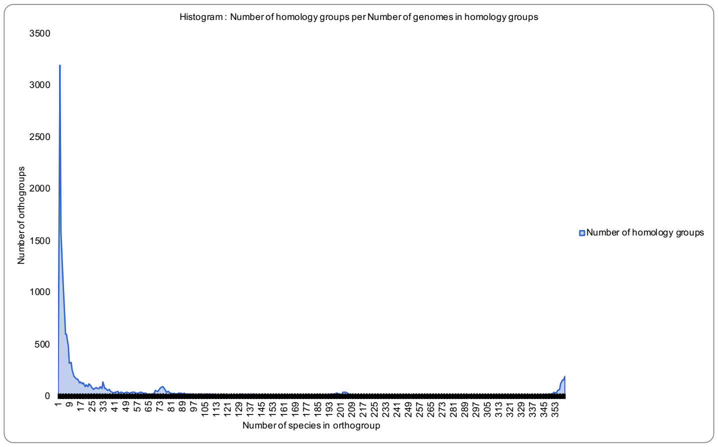

| | |
|---|---|
| 115 | 12 |
| 116 | 11 |
| 117 | 18 |
| 118 | 16 |
| 119 | 8 |
| 120 | 11 |
| 121 | 6 |
| 122 | 13 |
| 123 | 13 |
| 124 | 14 |
| 125 | 13 |
| 126 | 14 |
| 127 | 15 |
| 128 | 10 |
| 129 | 11 |
| 130 | 21 |
| 131 | 17 |
| 132 | 10 |
| 133 | 10 |
| 134 | 19 |
| 135 | 13 |
| 136 | 18 |
| 137 | 10 |
| 138 | 15 |
| 139 | 22 |
| 140 | 18 |
| 141 | 12 |
| 142 | 14 |
| 143 | 7 |
| 144 | 11 |
| 145 | 5 |
| 146 | 14 |
| 147 | 21 |
| 148 | 15 |
| 149 | 12 |
| 150 | 8 |
| 151 | 11 |
| 152 | 14 |
| 153 | 8 |
| 154 | 9 |
| 155 | 10 |
| 156 | 15 |
| 157 | 14 |
| 158 | 10 |
| 159 | 11 |
| 160 | 9 |
| 161 | 12 |
| 162 | 12 |
| 163 | 12 |
| 164 | 11 |
| 165 | 9 |
| 166 | 23 |
| 167 | 15 |
| 168 | 14 |
| 169 | 16 |
| 170 | 10 |
| 171 | 16 |
| 172 | 9 |
| 173 | 6 |
| 174 | 9 |
| 175 | 9 |
| 176 | 12 |
| 177 | 11 |
| 178 | 20 |
| 179 | 8 |
| 180 | 14 |
| 181 | 10 |
| 182 | 4 |
| 183 | 10 |
| 184 | 14 |
| 185 | 11 |
| 186 | 18 |
| 187 | 10 |
| 188 | 13 |
| 189 | 13 |
| 190 | 8 |
| 191 | 15 |
| 192 | 10 |
| 193 | 15 |
| 194 | 25 |
| 195 | 18 |
| 196 | 22 |
| 197 | 19 |
| 198 | 28 |
| 199 | 28 |
| 200 | 25 |
| 201 | 15 |
| 202 | 25 |
| 203 | 35 |
| 204 | 43 |
| 205 | 39 |
| 206 | 26 |
| 207 | 19 |
| 208 | 17 |
| 209 | 16 |
| 210 | 10 |
| 211 | 6 |
| 212 | 9 |
| 213 | 8 |
| 214 | 14 |
| 215 | 9 |
| 216 | 2 |
| 217 | 7 |
| 218 | 8 |
| 219 | 5 |
| 220 | 4 |
| 221 | 6 |
| 222 | 7 |
| 223 | 4 |
| 224 | 5 |
| 225 | 6 |
| 226 | 10 |
| 227 | 4 |
| 228 | 8 |
| 229 | 7 |
| 230 | 1 |
| 231 | 3 |
| 232 | 5 |
| 233 | 6 |
| 234 | 3 |
| 235 | 3 |
| 236 | 3 |
| 237 | 2 |
| 238 | 2 |
| 239 | 5 |
| 240 | 5 |
| 241 | 5 |
| 242 | 2 |
| 243 | 8 |
| 244 | 5 |
| 245 | 6 |
| 246 | 8 |
| 247 | 6 |
| 248 | 2 |
| 249 | 4 |
| 250 | 7 |
| 251 | 3 |
| 252 | 5 |
| 253 | 4 |
| 254 | 1 |
| 255 | 4 |
| 256 | 2 |

| | |
|---|---|
| 257 | 4 |
| 258 | 1 |
| 259 | 0 |
| 260 | 5 |
| 261 | 5 |
| 262 | 7 |
| 263 | 3 |
| 264 | 5 |
| 265 | 3 |
| 266 | 6 |
| 267 | 0 |
| 268 | 3 |
| 269 | 4 |
| 270 | 2 |
| 271 | 7 |
| 272 | 5 |
| 273 | 1 |
| 274 | 3 |
| 275 | 2 |
| 276 | 6 |
| 277 | 4 |
| 278 | 0 |
| 279 | 2 |
| 280 | 1 |
| 281 | 1 |
| 282 | 4 |
| 283 | 0 |
| 284 | 1 |
| 285 | 2 |
| 286 | 0 |
| 287 | 5 |
| 288 | 1 |
| 289 | 4 |
| 290 | 4 |
| 291 | 2 |
| 292 | 4 |
| 293 | 3 |
| 294 | 3 |
| 295 | 2 |
| 296 | 3 |
| 297 | 4 |
| 298 | 6 |
| 299 | 4 |
| 300 | 1 |
| 301 | 3 |
| 302 | 2 |
| 303 | 2 |
| 304 | 1 |
| 305 | 1 |
| 306 | 1 |
| 307 | 3 |
| 308 | 3 |
| 309 | 3 |
| 310 | 5 |
| 311 | 4 |
| 312 | 4 |
| 313 | 1 |
| 314 | 5 |
| 315 | 2 |
| 316 | 1 |
| 317 | 4 |
| 318 | 5 |
| 319 | 3 |
| 320 | 3 |
| 321 | 6 |
| 322 | 8 |
| 323 | 4 |
| 324 | 3 |
| 325 | 2 |
| 326 | 4 |
| 327 | 5 |
| 328 | 4 |
| 329 | 7 |
| 330 | 6 |
| 331 | 4 |
| 332 | 1 |
| 333 | 4 |
| 334 | 4 |
| 335 | 5 |
| 336 | 4 |
| 337 | 8 |
| 338 | 7 |
| 339 | 6 |
| 340 | 9 |
| 341 | 9 |
| 342 | 7 |
| 343 | 9 |
| 344 | 12 |
| 345 | 9 |
| 346 | 9 |
| 347 | 10 |
| 348 | 25 |
| 349 | 19 |
| 350 | 21 |
| 351 | 20 |
| 352 | 37 |
| 353 | 32 |
| 354 | 39 |
| 355 | 56 |
| 356 | 63 |
| 357 | 119 |
| 358 | 158 |
| 359 | 158 |
| 360 | 188 |
