# Supplementary material for: Reconstructing the Phylogeny of Corynebacteriales while Accounting for Horizontal Gene Transfer
Source: Genome Biol Evol. 2020 Apr 2;12(4):381–95. doi: 10.1093/gbe/evaa058 (PMC7186787; doi:10.1093/gbe/evaa058)
Supplement: evaa058_Supplementary_Data [file evaa058_supplementary_data.zip › Suplementary_Figures_S1-S7.pdf]

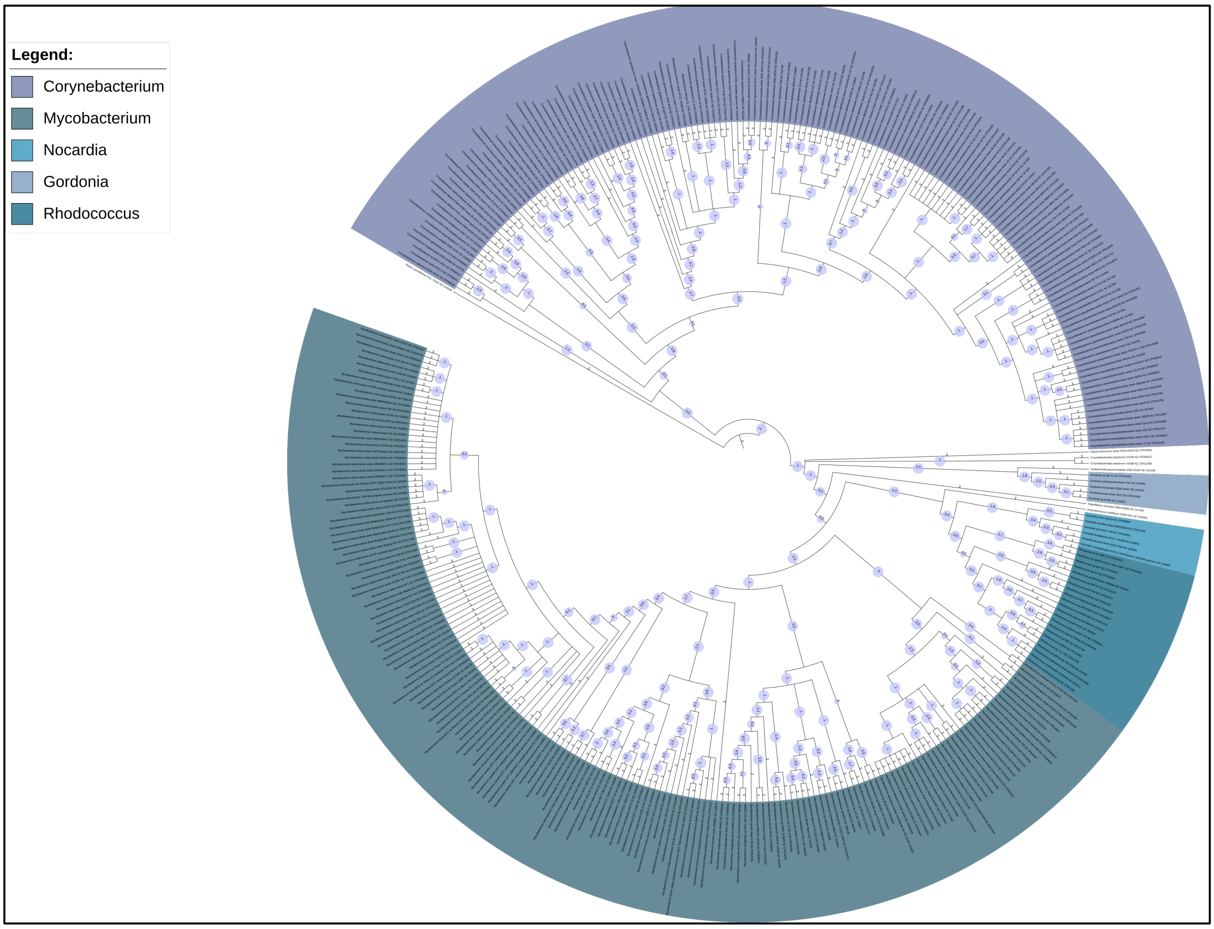

FIG. S1. Corynebacteriales preliminary species tree estimated using the concatenation of multiple sequence alignments of 13 putative orthology groups and RaXML (Stamatakis, 2014). The species belonging to the same genus are represented with the same color (Figure obtained using iTOL (Letunic and Bork, 2019)).

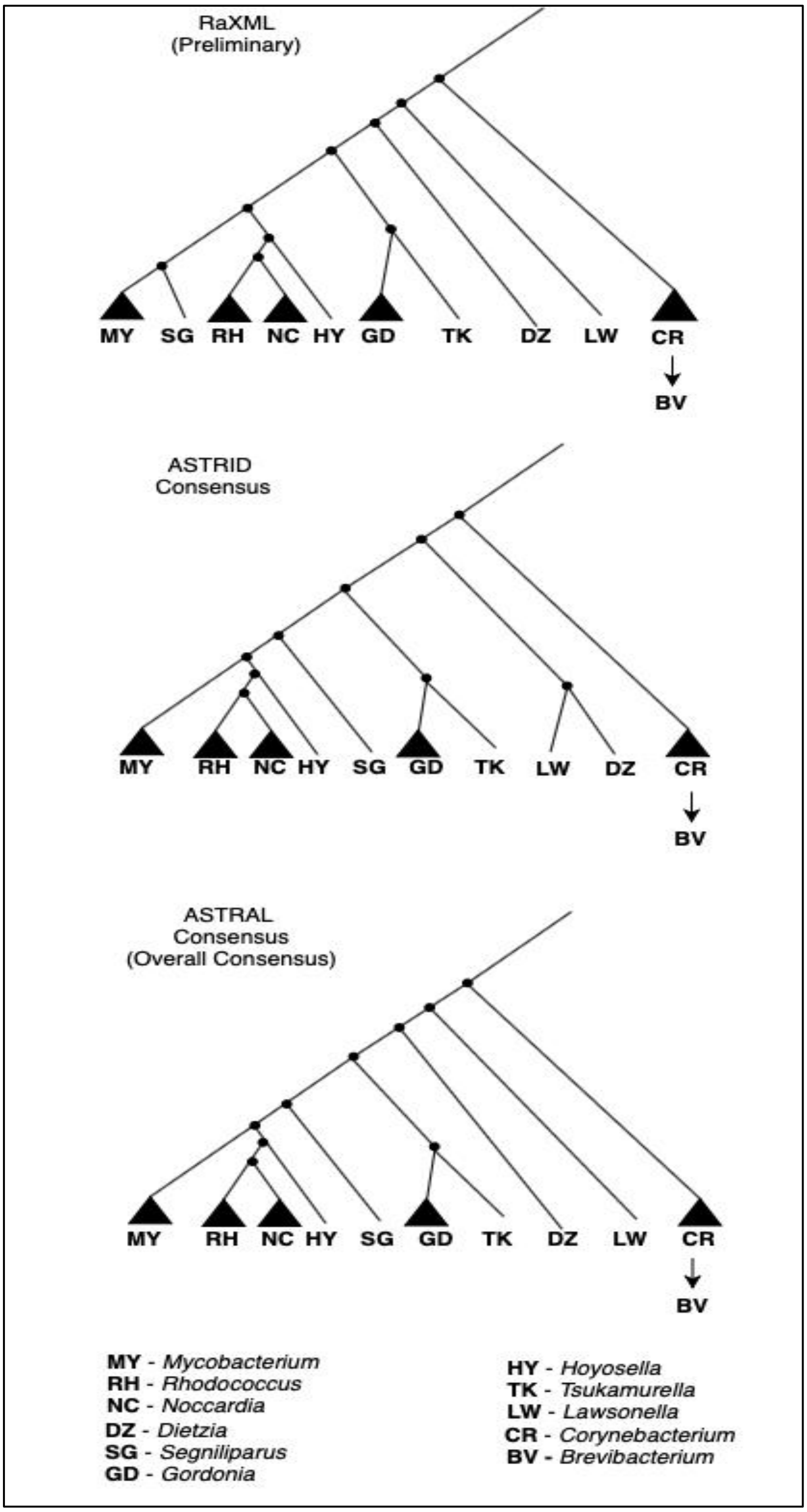

FIG. S2. Illustration, at the genus level, of the 4 consensus trees estimated in our work. A) phylogenomic tree reconstructed using the alignment of 13 single copy homology groups and RaXML; B) Consensus tree of ASTRID dataset; C) Consensus tree of ASTRAL dataset which is also the overall consensus between RaXML, ASTRID Consensus and ASTRAL Consensus. Genera for which the dataset contains more than one species are represented as triangles.

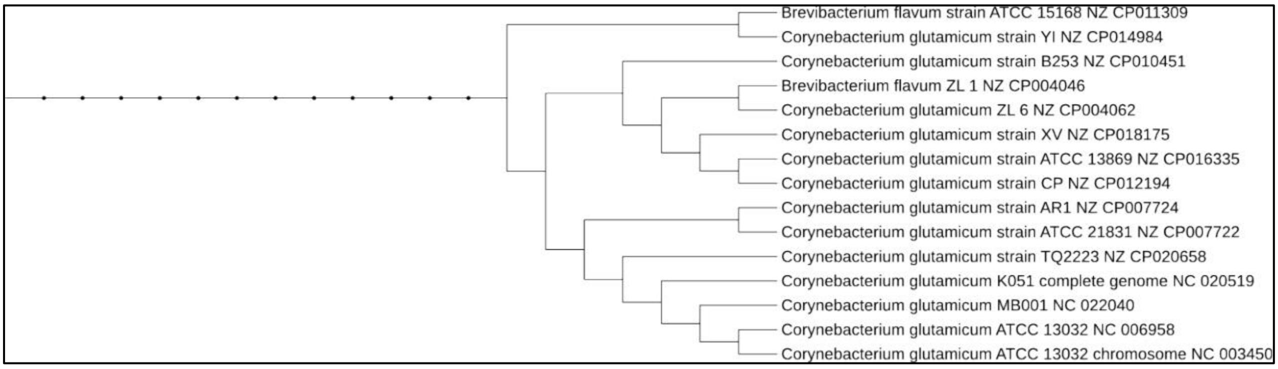

FIG. S3. Classification of *Brevibacterium* inside *Corynebacterium glutamicum*.

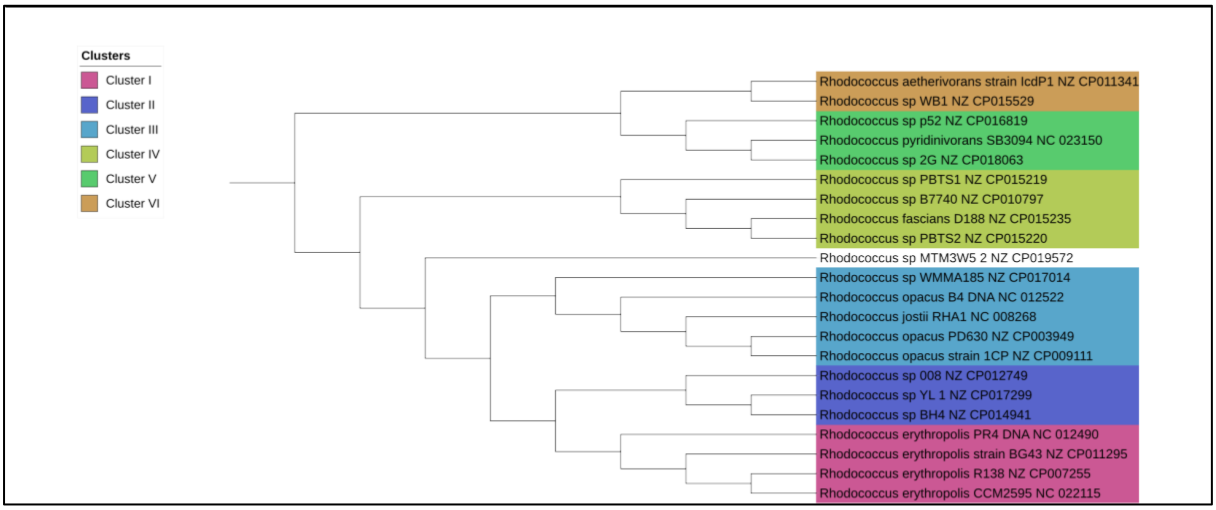

FIG. S4. Estimated phylogeny for *Rhodococcus* genus, with 6 clusters. Detailed method used to get this phylogeny (overall consensus tree: RaXML + ASTRID consensus+ ASTRAL consensus).

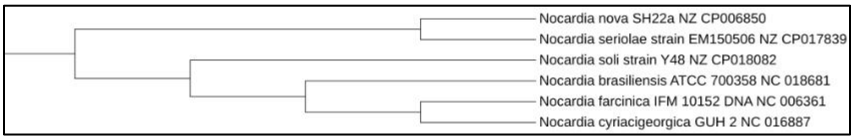

FIG. S5.. Estimated phylogeny for *Nocardia* genus.

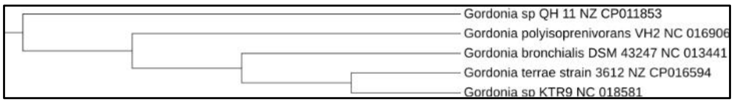

FIG. S6. Estimated phylogeny for Gordonia genus.

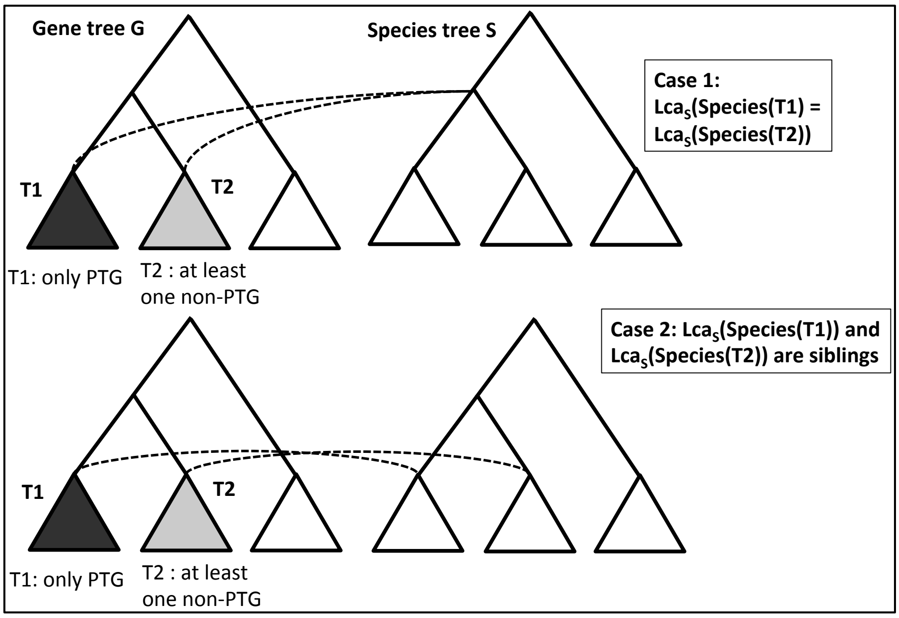

FIG. S7. Illustration of the phylogenetic method used in Step 5 to reclassify some putative transferred genes as vertically inherited genes. The two cases in which putative transferred genes are reclassified are depicted.
