# Supplementary material for: Reconstructing the Phylogeny of Corynebacteriales while Accounting for Horizontal Gene Transfer
Source: Genome Biol Evol. 2020 Apr 2;12(4):381–95. doi: 10.1093/gbe/evaa058 (PMC7186787; doi:10.1093/gbe/evaa058)
Supplement: evaa058_Supplementary_Data [file evaa058_supplementary_data.zip › Suplementary_Tables_S1-S3.pdf]

Table S1. Putative orthology groups used in Step 4 for computing the preliminary species tree

Group ID	Product
OG1103	50S Ribossomal protein L23
OG1104	50S Ribossomal protein L2
OG1105	30S Ribossomal protein S19
OG1106	50S Ribossomal protein L6
OG1107	50S Ribossomal protein L18
OG1108	30S Ribossomal protein S5
OG1113	ATP synthase subunit gamma
OG1116	phosphoglycerate kinase
OG1121	50S Ribossomal protein L35
OG1122	50S Ribossomal protein L20
OG1136	GTPase ObgE
OG1137	50S Ribosomal protein L27
OG1152	CarD TF regulator

Table S2. Number of gene trees in the 5 collections of trees considered in Step 5.

Max. number of missing genomes	Number of gene trees
72 (20%)	44
144 (40%)	46
180 (50%)	73
216 (60%)	93
288 (80%)	360

Table S3. Percentage of conserved clades between the consensus trees and the initial phylogenies reconstructed using ASTRID and ASTRAL.

	ASTRID 20	ASTRID 40	ASTRID 50	ASTRID 60	ASTRID 80
ASTRID Consensus	89.14	91.05	94.57	95.85	87.22

	ASTRAL 20	ASTRID 40	ASTRID 50	ASTRID 60	ASTRAL 80
ASTRAL Consensus	87.58	90.68	96.27	95.96	92.86
